# Genes Associated with Pancreas Development and Function Maintain Open Chromatin in iPSCs Generated from Human Pancreatic Beta Cells

**DOI:** 10.1016/j.stemcr.2017.09.020

**Published:** 2017-11-01

**Authors:** Matthias Thurner, Liraz Shenhav, Agata Wesolowska-Andersen, Amanda J. Bennett, Amy Barrett, Anna L. Gloyn, Mark I. McCarthy, Nicola L. Beer, Shimon Efrat

**Affiliations:** 1 The Wellcome Trust Centre for Human Genetics, University of Oxford, Oxford, UK; 2 Oxford Centre for Diabetes, Endocrinology and Metabolism, University of Oxford, Oxford, UK; 3 Department of Human Molecular Genetics and Biochemistry, Sackler School of Medicine, Tel Aviv University, Tel Aviv, Israel; 4 Oxford NIHR Biomedical Research Centre, Churchill Hospital, Oxford, UK

**Keywords:** human iPS cell, endoderm differentiation, diabetes, epigenetics, genomics, pancreas, beta-cell

## Abstract

Current *in vitro* islet differentiation protocols suffer from heterogeneity and low efficiency. Induced pluripotent stem cells (iPSCs) derived from pancreatic beta cells (BiPSCs) preferentially differentiate toward endocrine pancreas-like cells versus those from fibroblasts (FiPSCs). We interrogated genome-wide open chromatin in BiPSCs and FiPSCs via ATAC-seq and identified ∼8.3k significant, differential open chromatin sites (DOCS) between the two iPSC subtypes (false discovery rate [FDR] < 0.05). DOCS where chromatin was more accessible in BiPSCs (Bi-DOCS) were significantly enriched for known regulators of endodermal development, including bivalent and weak enhancers, and FOXA2 binding sites (FDR < 0.05). Bi-DOCS were associated with genes related to pancreas development and beta-cell function, including transcription factors mutated in monogenic diabetes (*PDX1*, *NKX2-2*, *HNF1A*; FDR < 0.05). Moreover, Bi-DOCS correlated with enhanced gene expression in BiPSC-derived definitive endoderm and pancreatic progenitor cells. Bi-DOCS therefore highlight genes and pathways governing islet-lineage commitment, which can be exploited for differentiation protocol optimization, diabetes disease modeling, and therapeutic purposes.

## Highlights

• Beta cells and fibroblasts were reprogrammed to pluripotent cells (BiPSCs/FiPSCs)
• ATAC-seq identified differential open chromatin sites in Bi-DOCS and Fi-DOCS
• Bi-DOCS were enriched for genes expressed in BiPSC-derived cells
• Bi-DOCS may explain the preferential differentiation of BiPSCs into islet cells

## Teaser

Beer and colleagues showed that iPSCs from beta cells (BiPSCs) preferentially differentiate into islet-lineage cells versus fibroblast iPSCs (FiPSCs). Differential open chromatin sites (DOCS) specific to BiPSCs (Bi-DOCS) were identified, enriched for pancreas development genes, and correlated with gene expression changes during directed differentiation. By governing developmental gene expression during differentiation, DOCS may explain the endodermal preference of BiPSCs and highlight novel beta-cell biology.

## Introduction

Human pancreatic islets have been placed center stage in type 2 diabetes pathogenesis (Dimas et al., 2014). Current disease-modeling efforts are often frustrated by the limited availability of human physiologically authentic islet-like cells. Derivation of endocrine pancreas from iPSCs represents one solution for generating sufficient numbers of physiologically and disease-relevant human islet-like cells (Nostro et al., 2015, Pagliuca et al., 2014, Rezania et al., 2014). While directed *in vitro* differentiation of iPSCs routinely yields cells positive for islet hormones, such as insulin and glucagon, these cell populations are heterogeneous, contain poly-hormonal cells, and are functionally immature versus primary human islets (Rezania et al., 2014, van de Bunt et al., 2016). Differentiation efficiency also varies across iPSC lines (Bar-Nur et al., 2011, Burrows et al., 2016, Kim et al., 2010, Kyttala et al., 2016, Polo et al., 2010, Rouhani et al., 2014).

Possible causes for inconsistencies in differentiation capacity include technical factors such as reprogramming strategy (Balboa and Otonkoski, 2015), but also line-specific characteristics such as donor genotype (Burrows et al., 2016, Kyttala et al., 2016, Rouhani et al., 2014). There is also evidence to support an epigenetic “memory” in iPSCs (Bar-Nur et al., 2011, Kim et al., 2010, Polo et al., 2010), this comprising epigenomic and transcriptomic signatures of the original reprogrammed cell type, which may erode over prolonged periods of passaging in culture (Bar-Nur et al., 2011, Kim et al., 2010, Polo et al., 2010).

The epigenome plays an important role in establishing developmental competence in stem cells (Wang et al., 2015, Xie et al., 2013). Epigenetic memory could thus account for the enhanced propensity of beta-cell-derived iPSCs (BiPSCs) to differentiate down the endocrine pancreas lineage versus those derived from skin fibroblasts (FiPSCs; Bar-Nur et al., 2011). Here, we aimed to capitalize upon proposed BiPSC epigenetic memory to identify genes and pathways governing islet development and identity. By utilizing an assay for transposase accessible chromatin with high-throughput sequencing (ATAC-seq), we first aimed to define the open chromatin landscape in BiPSCs. Secondly, by comparison with global open chromatin in FiPSCs, we aimed to identify BiPSC-specific differential open chromatin sites (Bi-DOCS). We then integrated Bi-DOCS with publicly available genomic annotations to highlight regulatory elements, genes, and pathways that may explain preferential differentiation of BiPSCs toward endocrine pancreas-like cells.

Finally, to confirm that differences in open chromatin lead to changes in gene expression, we compared the transcriptome of cells derived from directed differentiation of both BiPSCs and FiPSCs toward two key stages of islet development (definitive endoderm and pancreatic progenitors). Our study improves understanding of human islet development, which may provide clues for improving *in vitro* differentiation protocols, facilitate more advanced disease modeling, and eventually contribute to therapeutic applications (Rezania et al., 2014, ViaCyte, 2014).

## Results

### Mapping the Open Chromatin Landscape in BiPSCs

We generated five BiPSC lines from three independent non-diabetic donors and five FiPSC lines from two independent non-diabetic donors using reprogramming strategies described previously (Bar-Nur et al., 2011, van de Bunt et al., 2016) (Table S1). Of note, because of differences in source, the BiPSCs were on average ten passages lower than the FiPSC lines. All iPSC lines passed quality control assessing pluripotency and differentiation capacity (Figures S1A–S1D). All but two lines (BiPSC-D1 and D2, Table S1) were karyotypically normal; however, as shown in the section on “*In Silico* and Cellular Validation of DOCS”, removing these two lines did not substantially alter the results reported in this study. As shown previously (Bar-Nur et al., 2011), BiPSCs showed enhanced spontaneous *in vitro* differentiation capacity into islet-lineage cells expressing *FOXA2*, *PDX1*, and *INS,* compared with FiPSCs (Figure S1E).

**Figure 1:**
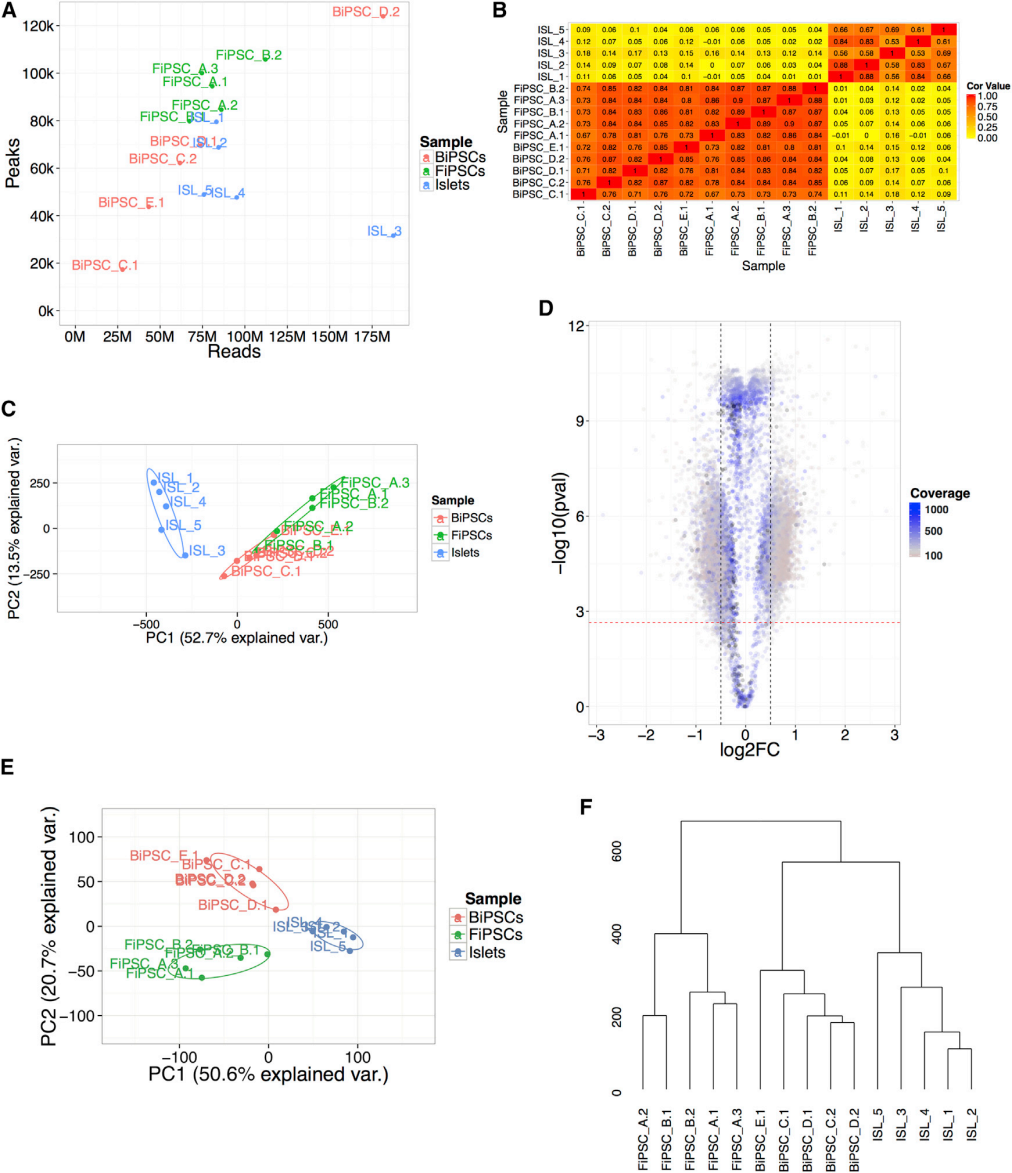
The Open Chromatin Landscape of BiPSCs and FiPSCs (A) Sample read (x axis) and peak number (y axis). (B) Heatmap with mean sample correlation (rho) across peaks. (C) Sample PCA plot based on peak read depth. (D) DOCS Volcano plot with log2FC (x axis) and −log10 p value (y axis). DOCS, dashed black (log2FC ± 0.5) and red (FDR < 0.05) lines. (E) Sample PCA plot based on DOCS read depth. (F) Hierarchical clustering of samples based on DOCS read depth. Color of cell type in (A, C, and E), BiPSCs (red), FiPSCs (green), human islets (blue).

We performed ATAC-seq in all ten lines, as well as in five adult human islet samples. We generated between 28 and 186 million mapped and filtered reads per sample (Figure 1A), and identified between 17.3k and 123.9k open chromatin peaks (false discovery rate [FDR] < 0.01). Consistent with the pluripotent nature of both iPSC subtypes, we found the open chromatin pattern to be highly similar between BiPSCs and FiPSCs (median rho = 0.84, Figure 1B), and clearly distinct from primary human islets. Principal component analysis (PCA) across open chromatin peaks of all samples also confirmed that the two iPSC types were highly similar, and that iPSCs did not cluster by donor genotype (Figure 1C).

### Identifying BiPSC-Specific Open Chromatin

Despite the similarities in open chromatin between the two iPSC subtypes, we were able to identify differential open chromatin sites (DOCS) between BiPSCs and FiPSCs (Supplemental Experimental Procedures). We found 8.3k significant DOCS (FDR < 0.05) with a minimum absolute log2 fold change (log2FC) of 0.5 (Figure 1D). About 4.8k (58%) of DOCS were characterized by an increase in open chromatin in BiPSCs (Bi-DOCS), while the remaining 3.5k sites were more open in FiPSCs (Fi-DOCS). PCA and hierarchical clustering of normalized ATAC-seq read depth across DOCS showed that BiPSCs were more similar to human islets than FiPSCs (Figures 1E and 1F). Again, we did not observe sample clustering by donor. This suggests that genetic variation is not the predominant driver of differences in open chromatin in our study.

### Bi-DOCS Are Enriched in Chromatin States Involved in Developmental Competence

**Figure 2:**
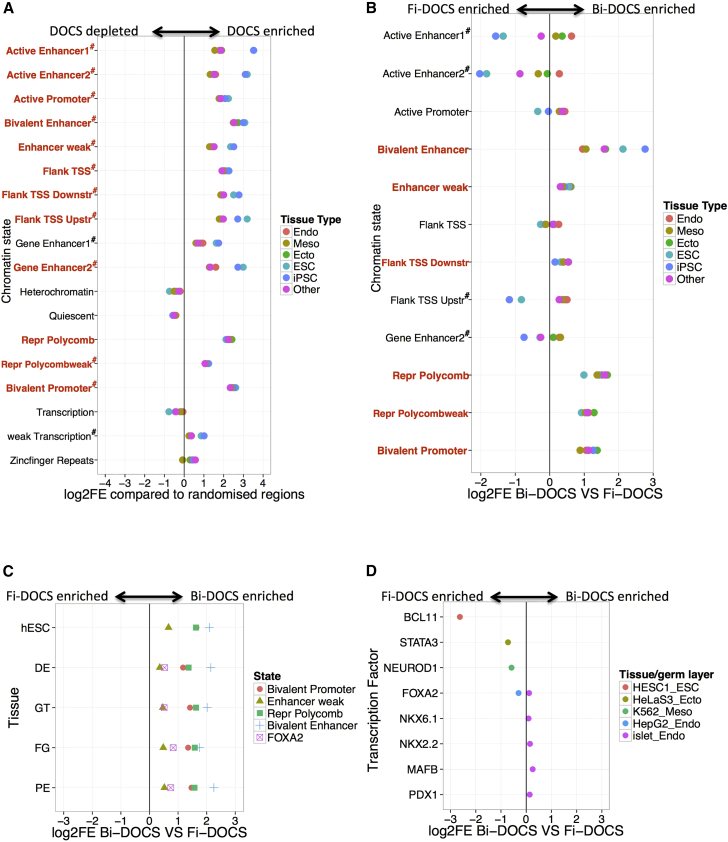
Enrichment of Bi-DOCS and Fi-DOCS in Chromatin States (A) Enrichment (x axis) of DOCS in Epigenome Roadmap chromatin states (y axis) grouped according to pluripotent cell/germ layer type. Bold red states were enriched across all germ layer and pluripotent stem cell states while # indicates that ESC/iPSC states had highest enrichment. (B) Log2FE ratio (x axis) of Bi-DOCS versus Fi-DOCS across chromatin states (y axis) enriched in (A). Bold red states are enriched for Bi-DOCS while # shows states most enriched for Fi-DOCS in ESC/iPSC lines. (C) Log2FE ratio (x axis) of Bi-DOCS versus Fi-DOCS across chromatin states and FOXA2 TFBS (shape/color) identified during different stages (y axis) of endodermal development. human stem cells (hESC, FOXA2 not available), definitive endoderm (DE), gut tube (GT), fore gut (FG), and pancreatic endoderm (PE). (D) Log2FE ratio (x axis) of Bi-DOCS versus Fi-DOCS across HESC-H1 (stem cell, red), HeLaS3 (ectoderm, dark yellow), K562 (mesoderm, green), HepG2 (endoderm, blue), and islet (endoderm, purple) TFBS (y axis).

To uncover the potential regulatory landscape of DOCS, we obtained data on 18 predefined chromatin states from 98 Epigenome Roadmap cell types, which include human islets (Kundaje et al., 2015). Samples were grouped according to pluripotency (iPSCs and embryonic stem cells [ESCs], cell types per group = 4–5), germ layer (mature cells and tissues, cell types per group = 17–42), or “other” (germ layer status unclear, cell type number = 10). We found through permutation that DOCS were significantly enriched in enhancer, promoter, flanking transcription start site (TSS) and polycomb-repressed chromatin states, compared with random regions (mean FDR adjusted p value across all cell types of a given chromatin and germ layer/stem cell type < 0.05 and log2 fold enrichment (log2FE) > 0; Figures 2A and S2A).

We observed that across groups Bi-DOCS were enriched for bivalent enhancers, bivalent promoters, weak enhancers, flanking TSS downstream elements, and polycomb-repressed regions versus Fi-DOCS (mean log2FE ratio of Bi-DOCS versus Fi-DOCS (log2FE ratio) > 0, Figure 2B). In contrast, Fi-DOCS showed significant enrichment in ESC and iPSC active enhancer chromatin states compared with Bi-DOCS (log2FE ratio < 0, Figure 2B). These results show that Bi-DOCS overlap important regulatory elements known to be involved in developmental competence, including endoderm developmental competence (weak enhancer and bivalent states; Wang et al., 2015, Xie et al., 2013).

### Bi-DOCS Are Enriched for Regulators of Early Endodermal Lineage Commitment

Endoderm developmental competence is regulated by epigenetic factors, including weak enhancers (marked by H3K4me1), bivalent regions marked by H3K27me3 (bivalent enhancers/promoters), and changes in gene expression governed by lineage-specific transcription factors such as FOXA2 (Wang et al., 2015, Xie et al., 2013). To confirm whether Bi-DOCS were enriched for such early endodermal regulatory states, we identified FOXA2 transcription factor binding sites (TFBS) and chromatin regulatory states across discrete stages of a previously published model of pancreatic endoderm development (Figure S2B; Supplemental Experimental Procedures) (Wang et al., 2015, Xie et al., 2013). We found that Bi-DOCS (versus Fi-DOCS) showed significant enrichment in weak enhancers, bivalent enhancers, bivalent promoters, polycomb-repressed regions, and FOXA2 TFBS as identified across all stages of pancreatic endoderm development (FDR < 0.05, log2FE ratio > 0, Figures 2C and S2C). Enrichment for these regulatory regions in Bi-DOCS may explain the preferential endodermal lineage commitment of BiPSCs.

### Bi-DOCS Enrichment Is Specific to Pancreatic Endoderm Lineage Commitment

To show that Bi-DOCS are specifically enriched for pancreatic endoderm regulatory annotations and not regulatory annotations in other cell types or mature islets, we obtained publicly available TFBS information for four Encode cell lines (representative of pluripotency or germ layer commitment) (Encode Project Consortium, 2012) and TFBS active in human islets (Pasquali et al., 2014; Figures 2D and S2D).

While we found strong enrichment of all DOCS in all TFBS (Figure S2D), Bi-DOCS (compared with Fi-DOCS) were not enriched for TFBS from any of the four tested cell lines (Figure 2D), and showed only weak global enrichment in TFBS from adult primary human islets (max log2FE ratio = 0.3; Figures 2D and S2D).

These data confirm that Bi-DOCS are highly and specifically enriched for TFBS and chromatin states active in early pancreatic endoderm development (see previous section). However, despite this global pattern, we also found a number of Bi-DOCS associated with mature beta-cell genes including *INS* and *PDX1* as described below.

### Bi-DOCS Are Enriched for Genes and Pathways Regulating Pancreatic Islet Development and Function

To understand which genes and pathways are regulated by regions mapping to Bi-DOCS, we conducted a pathway enrichment test (Supplemental Experimental Procedures). We found that the 4.8k Bi-DOCS were significantly enriched in 27 Gene Ontology (GO) Biological Process and 2 MSigDB Pathway terms (binomial and hypergeometric FDR < 0.05 and binomial region fold enrichment and minimum gene enrichment > 2; Tables 1 and S2). Stratification of Bi-DOCS into different regulatory annotations highlighted additional terms in which Bi-DOCS were significantly enriched (hypergeometric FDR < 0.05 and enrichment > 2, compared with background; Tables S3 and S4, Supplemental Experimental Procedures). These include diabetes-relevant terms such as maturity-onset diabetes of the young (MODY), a form of monogenic diabetes caused mainly by mutations in islet transcription factors (Murphy et al., 2008). In addition, terms associated with glucose sensing and insulin metabolism and secretion were identified, as well as those relating to endocrine pancreas fate decisions and beta-cell development. For instance, *FOXA2* (Lee et al., 2002), *NKX2-2* (Sussel et al., 1998), and *PDX1* (Stoffers et al., 1997) were highlighted, each of these genes serving important roles in islet development. WNT and NOTCH signaling terms were also enriched; these pathways are important in the induction of posterior endoderm and pancreatic progenitors, as well as pancreatic endocrine versus exocrine fate choices (Cras-Meneur et al., 2009, Nostro et al., 2011). Multiple terms relating to neuronal development were also enriched; this is consistent with gene expression profiles observed between developing pancreatic islets and neurons (van Arensbergen et al., 2010).

**Table 1:** Bi-DOCS-Associated GO Biological Processes and MSigDB Pathways Terms

Term	Pathway Description	DOCS Trend	Gene Number	Region Enrichment	Gene Enrichment	Regional FDR	Gene FDR	Associated Function
GO	neural tube patterning	Bi-DOCS	80	3.27	2.69	8.7 × 10^−17^	2.4 × 10^−5^	neuro-developmental
GO	positive regulation of embryonic development	Bi-DOCS	51	4.75	3.23	1.0 × 10^−16^	9.3 × 10^−3^	developmental
GO	non-canonical Wnt receptor signaling pathway	Bi-DOCS	65	3.55	2.10	2.4 × 10^−15^	3.9 × 10^−2^	WNT/NOTCH signaling
GO	hindbrain morphogenesis	Bi-DOCS	82	2.84	2.32	4.1 × 10^−14^	4.1 × 10^−4^	neuro-developmental
GO	branching involved in mammary gland duct morphogenesis	Bi-DOCS	68	3.20	2.53	5.2 × 10^−14^	3.0 × 10^−3^	developmental
GO	cerebellum morphogenesis	Bi-DOCS	75	2.86	2.36	4.9 × 10^−13^	6.8 × 10^−4^	neuro-developmental
GO	mammary gland duct morphogenesis	Bi-DOCS	94	2.39	2.47	6.2 × 10^−12^	1.8 × 10^−4^	developmental
GO	mammary gland epithelium development	Bi-DOCS	123	2.10	2.12	9.1 × 10^−12^	1.4 × 10^−4^	developmental
GO	dorsal spinal cord development	Bi-DOCS	51	3.42	2.57	1.4 × 10^−11^	3.8 × 10^−3^	neuro-developmental
GO	cerebellum development	Bi-DOCS	110	2.10	2.02	1.4E-10	1.1 × 10^−4^	neuro-developmental
GO	cerebellar cortex morphogenesis	Bi-DOCS	58	2.91	2.31	1.9E-10	6.6 × 10^−3^	neuro-developmental
GO	spinal cord association neuron differentiation	Bi-DOCS	38	3.83	2.69	5.6E-10	1.6 × 10^−2^	neuro-developmental
GO	dorsal/ventral neural tube patterning	Bi-DOCS	50	2.99	2.83	2.0 × 10^−9^	9.5 × 10^−4^	neuro-developmental
GO	negative regulation of Notch signaling pathway	Bi-DOCS	34	3.73	2.55	1.1 × 10^−8^	1.0 × 10^−2^	WNT/NOTCH signaling
GO	cell differentiation in spinal cord	Bi-DOCS	93	2.07	2.83	1.1 × 10^−8^	2.6 × 10^−9^	neuro-developmental
GO	cell differentiation in hindbrain	Bi-DOCS	55	2.61	2.64	2.4 × 10^−8^	1.6 × 10^−3^	neuro-developmental
GO	cerebellar cortex development	Bi-DOCS	65	2.38	2.02	2.8 × 10^−8^	8.8 × 10^−3^	neuro-developmental
GO	somitogenesis	Bi-DOCS	93	2.00	2.05	4.6 × 10^−8^	4.1 × 10^−4^	developmental
GO	white fat cell differentiation	Bi-DOCS	29	3.37	2.69	1.3 × 10^−6^	4.5 × 10^−2^	developmental
GO	embryonic axis specification	Bi-DOCS	65	2.10	2.02	2.0 × 10^−6^	1.9 × 10^−2^	developmental
GO	ventral spinal cord interneuron fate commitment	Bi-DOCS	34	2.91	3.67	2.8 × 10^−6^	3.1 × 10^−4^	neuro-developmental
GO	regulation of insulin receptor signaling pathway	Bi-DOCS	44	2.44	2.02	5.0 × 10^−6^	3.3 × 10^−2^	diabetes/insulin
GO	positive regulation of focal adhesion assembly	Bi-DOCS	25	3.46	2.80	5.7 × 10^−6^	1.9 × 10^−2^	other
GO	cerebellar cortex formation	Bi-DOCS	40	2.54	2.43	6.4 × 10^−6^	1.8 × 10^−2^	neuro-developmental
GO	regulation of Notch signaling pathway	Bi-DOCS	58	2.06	2.21	1.4 × 10^−5^	1.1 × 10^−3^	WNT/NOTCH signaling
GO	spinal cord dorsal/ventral patterning	Bi-DOCS	39	2.34	2.55	4.3 × 10^−5^	1.0 × 10^−2^	neuro-developmental
GO	ventral spinal cord interneuron differentiation	Bi-DOCS	35	2.46	3.42	5.0 × 10^−5^	4.1 × 10^−4^	neuro-developmental
MSigDB pathway	Wnt signaling network	Bi-DOCS	53	2.68	2.31	9.4 × 10^−8^	2.8 × 10^−2^	WNT/NOTCH signaling
MSigDB pathway	maturity-onset diabetes of the young	Bi-DOCS	40	2.15	2.43	3.0 × 10^−4^	2.5 × 10^−2^	diabetes/insulin

To evaluate signatures of epigenetic memory, which could explain the enhanced endodermal differentiation capacity of BiPSCs, we tested whether Bi-DOCS-associated genes relevant for endodermal development were also open in human islets. We found that 99% of Bi-DOCS-associated genes, which are specifically expressed during endocrine pancreas development (van de Bunt et al., 2016), were also associated with open chromatin in human islets. In addition, when comparing the total number of Bi-DOCS and adult islet open chromatin-associated genes, we found that Bi-DOCS-associated genes were significantly enriched in stage-specific endocrine pancreas developmental genes versus those associated with adult islet open chromatin (Fisher test odds ratio [OR] = 1.3, p = 1.9 × 10^−13^).

The overlap of Bi-DOCS-associated genes with those open in human islets suggests BiPSCs could retain an epigenetic signature of their cell type of origin. In addition, the enrichment of Bi-DOCS for endocrine pancreas developmental stage-specific genes could explain the enhanced propensity of BiPSCs to differentiate down this same developmental lineage.

### *In Silico* and Cellular Validation of DOCS

**Figure 3:**
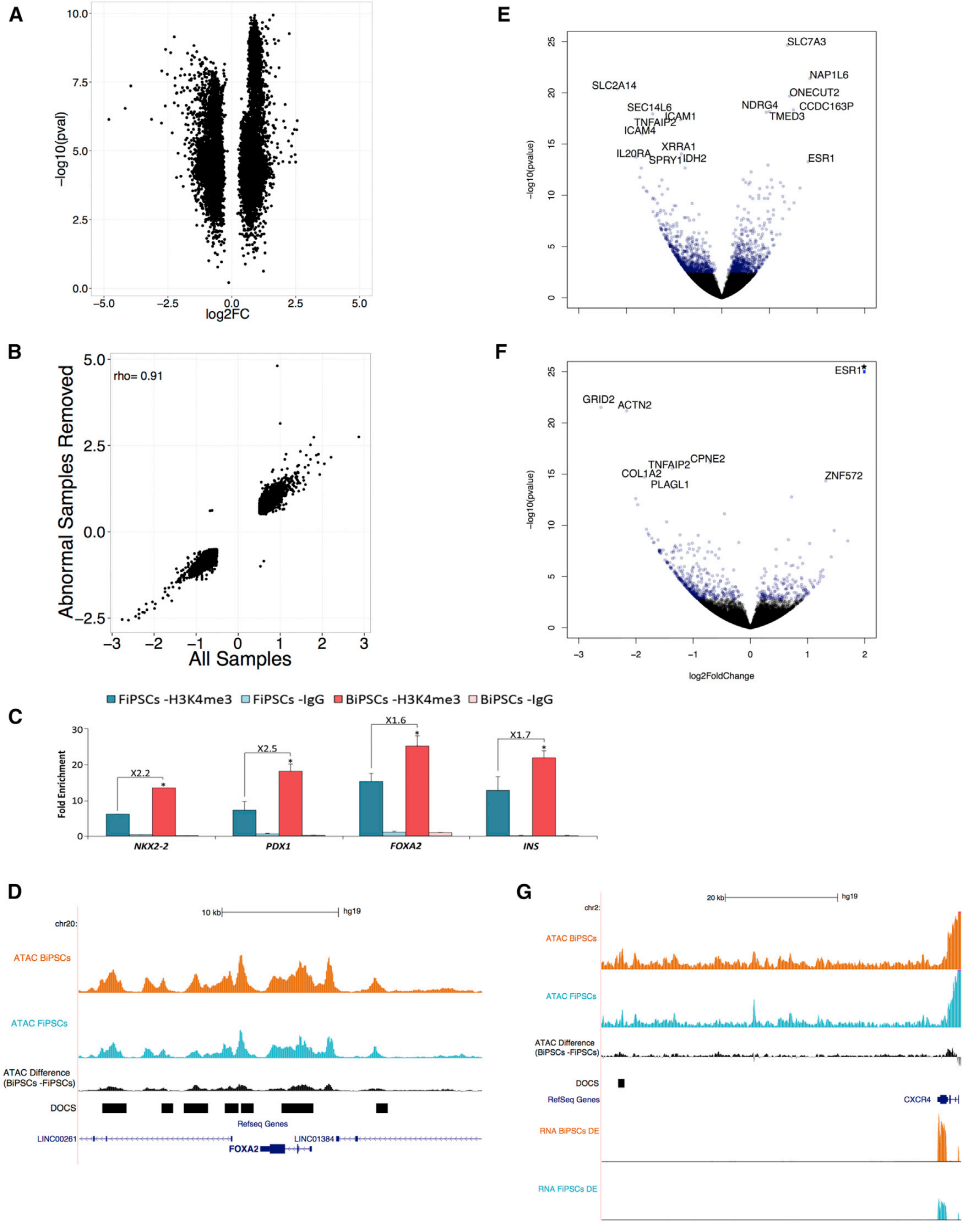
Validation of DOCS (A) DOCS Volcano plot with log2FC (x axis) and −log10 p value (y axis) of karyotypically normal samples only. (B) Log2FC correlation of overlapping DOCS identified from all samples (x axis) or only karyotypically normal samples (y axis). (C) ChIP-qPCR analysis of H3K4me3 in BiPSCs (n = 5) and FiPSCs (n = 4). Values are means ± SEM and normalized to *CRYAA* and *TEX15*. ^∗^p < 0.05. (D) *FOXA2* region with normalized read depth of BiPSCs (orange) and FiPSCs (blue) samples. In black, ATAC read depth difference between iPSC types. Bottom tracks: DOCS (black bar) and genes (blue). (E and F) Volcano plot with log2FC (x axis) and −log10 p value (y axis) of differentially expressed genes between FiPSC- and BiPSC-derived DE (E) and PP (F) cells. ^∗^*ESR1* at PP stage out-of-scale in (F) (true log2FC = 5.74, −log10 p value = 100.9). (G) *CXCR4* region comparing normalized ATAC-seq read depth of BiPSCs (orange) and FiPSCs (blue, top). In black (middle), read depth difference between BiPSCs and FiPSCs. DOCS (black bar) are shown in the middle. Bottom tracks: *CXCR4* RNA-seq data of BiPSC- (orange) and FiPSC (blue)-derived DE cells.

Using random permutations of sample labels to account for biases caused by donor-dependent factors, we found that the number of DOCS identified in permuted samples was lower than that observed in the original analysis (mean random sites = 2.0k versus 8.3k in the original analysis). This confirms that donor-specific factors are not the major driver in the differential open chromatin analysis in our study. Furthermore, we found that removing samples with an abnormal karyotype (lines BiPSC-D 1–2) yielded results similar to the original analysis (at 4.8k/8.3k overlapping DOCS) including all samples (Figures 3A and 3B, Supplemental Experimental Procedures).

ATAC-seq data and prediction of Bi-DOCS were validated *in vitro* using H3K4me3 chromatin immunoprecipitation (ChIP)-qPCR and primers flanking a subset of DOCS near the promoters of *PDX1*, *NKX2-2*, *FOXA2*, and *INS*; these genes highlighted by the gene enrichment analysis. All five BiPSC lines showed higher chromatin enrichment in the promoter regions of *PDX1*, *NKX2-2*, *FOXA2,* and *INS* versus FiPSCs (Figure 3C). Figure 3D visualizes seven DOCS between FiPSCs and BiPSCs around the *FOXA2* promoter.

### Bi-DOCS Affect Gene Expression at Key Stages of Islet Development

To confirm that Bi-DOCS contain regulatory annotations that have an impact on gene expression during development, we performed directed differentiation of a subset of FiPSCs and BiPSCs cell lines toward islet-like cells and collected RNA at two key developmental stages: definitive endoderm (DE) and pancreatic progenitors (PP) (Table S1). RNA-seq and differential expression analysis identified 1,247 protein-coding genes differentially expressed (FDR < 0.05) between BiPSCs- and FiPSCs-derived cells at the DE stage (567 genes upregulated in BiPSC-derived and 680 in FiPSC-derived cells, Figure 3E), and 607 genes at the PP stage (181 genes upregulated in BiPSC-derived cells, 426 in FiPSC-derived cells, Figure 3F). Genes upregulated in BiPSCs-derived cells at both stages were significantly enriched in those mapping to Bi-DOCS (hypergeometric FDR_DE_ = 3.4 × 10^−7^, FDR_pp_ = 9.1 × 10^−4^), while genes upregulated in FiPSC-derived cells were significantly enriched in Fi-DOCS genes (hypergeometric FDR_DE_ = 2.5 × 10^−14^, FDR_pp_ = 5.0 × 10^−8^, Table S6). We detected a proximal Bi-DOCS site for 126 of the 567 genes upregulated at BiPSC-derived DE, and a Fi-DOCS site for 145 of the 680 genes upregulated at FiPSC-derived DE. Bi-DOCS-associated genes include those of known endoderm developmental biology, such as *CXCR4* (Katsumoto and Kume, 2011). *CXCR4* was significantly upregulated in BiPSCs at the DE stage (log2FC = 1.01, p_adj_ = 7.4 × 10^−6^, Figure 3G) and has a Bi-DOC site that overlaps a DE weak enhancer; a type of chromatin state involved in endodermal development (Wang et al., 2015).

Finally, genes upregulated in BiPSCs at the DE stage were significantly enriched for FOXA2 target genes expressed at this stage (hypergeometric FDR = 1.4 × 10^−53^, Table S6) which is in line with the FOXA2 TFBS enrichment in Bi-DOCS.

## Discussion

Here, we systematically cataloged sites of open chromatin across the genome of BiPSCs to explain the preferential endodermal lineage commitment of BiPSCs versus FiPSCs. We were able to identify Bi-DOCS and showed that these Bi-DOCS were enriched for genomic regulatory annotations such as weak enhancers, bivalent enhancers, bivalent promoters, and FOXA2 binding sites shown to be active during endoderm lineage commitment (Wang et al., 2015, Xie et al., 2013).

We also confirmed that Bi-DOCS could be linked to gene expression changes at two stages of *in vitro* differentiation into islet-like cells. We also observed a significant enrichment of the FOXA2 targets reported at the DE stage within the genes upregulated in BiPSCs at this stage. FOXA2 is one of the master regulators of endodermal development (Raum et al., 2006), with FOXA2 binding sites being enriched at endodermal poised weak enhancers, thereby priming cells to receive extracellular cues for differentiation into endodermal lineage organs (Wang et al., 2015). Bi-DOCS were also enriched for GO terms relating to early pancreatic endoderm signaling pathways (Nostro et al., 2015, Pagliuca et al., 2014, Rezania et al., 2014), as well as MODY genes. This suggests that Bi-DOCS represent signatures of epigenetic memory and drive the differences in gene expression, which may account for the enhanced propensity of BiPSCs to differentiate toward endodermal lineage. Bi-DOCS may thus provide clues to genes and pathways involved in islet-lineage development.

The extent of epigenetic “memory” in reprogrammed iPSCs remains a subject of debate. Alongside data suggesting donor genotype may drive differentiation potential (Burrows et al., 2016, Kyttala et al., 2016, Rouhani et al., 2014), technical artifacts such as culture conditions, cell-cycle phase, and reprogramming technology may also give rise to transcriptional and epigenetic differences between iPSC lines. Additional work will be required to determine whether the differences reported here are solely dependent on the donor cell type. Specifically, the two iPSC types in our study differ substantially in passage number, and prolonged passaging has been suggested to eradicate iPSC epigenetic memory (Polo et al., 2010).

Future work should focus on increasing sample number and integrating our data with additional genomic annotations to further elucidate the mechanisms driving developmental competence and differentiation capacity. In conclusion, our findings provide a valuable resource for improving endocrine pancreas lineage differentiation protocols and may lead to the development of enhanced islet cell models, and ultimately improved therapeutic approaches for diabetes.

## Experimental Procedures

### iPSC Generation and Human Islet Sample Collection

As described in Supplemental Experimental Procedures, relevant Ethics and Institutional Review Boards (IRBs) approved the use of human islets and human iPSCs in this study. BiPSC and FiPSC lines were generated by reprogramming beta cells and skin fibroblasts as described previously (Bar-Nur et al., 2011, van de Bunt et al., 2016). Five human islets were freshly isolated from cadaveric donors as previously described (van de Bunt et al., 2016). All samples were processed for ATAC-seq as described in the Supplemental Experimental Procedures.

### Computational and Statistical Analysis

ATAC-seq and RNA-seq reads were processed and aligned to the genome (build hg19). We then predicted open chromatin regions, identified DOCS, and differentially expressed genes as described in the Supplemental Experimental Procedures. Statistical analysis was performed in R version 3.0.2 unless stated otherwise. Data have been deposited in public repositories (Supplemental Experimental Procedures).

## Author Contributions

M.T., L.S., A.L.G., M.I.M., N.L.B., and S.E. designed the study. A.B. and A.J.B. obtained the human islet samples and performed quality control. L.S. and N.L.B. performed iPSC experiments. M.T. performed bioinformatics analysis of ATAC-seq data. A.W.-A. analyzed the RNA-seq data. M.T., L.S., and N.L.B. wrote the manuscript. A.W.A., M.I.M., A.L.G., and S.E. gave conceptual advice and edited the manuscript.

## Accession Numbers

Data have been deposited at the EBI hosted European Genome-phenome Archive (EGA, http://www.ebi.ac.uk/ega/) and European Nucleotide Archive (ENA, http://www.ebi.ac.uk/ena). EGA: EGAS00001002591 (islet ATAC-seq and iPSC RNA-seq). ENA: PRJEB21856 (iPSC ATAC-seq).

## Supplemental Information

Document S1. Supplemental Experimental Procedures, Figures S1 and S2, and Tables S1, S5, and S6

Table S2. GO Biological Processes and MSigDB Pathway Terms Associated Gene Names, Related to Table 1

Table S3. GO Biological Processes and MSigDB Pathway Terms Associated with Bi-DOCS Overlapping hESC H1 Bivalent Enhancer Chromatin States, Related to Table 1

Table S4. GO Biological Processes and MSigDB Pathways Terms Associated with Bi-DOCS Overlapping hESC H1 Bivalent Promoter Chromatin States, Related to Table 1

Document S2. Article plus Supplemental Information
