# Supplementary material for: Genes Associated with Pancreas Development and Function Maintain Open Chromatin in iPSCs Generated from Human Pancreatic Beta Cells
Source: Stem Cell Reports. 2017 Nov 1;9(5):1395–405. doi: 10.1016/j.stemcr.2017.09.020 (PMC5831005; doi:10.1016/j.stemcr.2017.09.020)
Supplement: Document S1. Supplemental Experimental Procedures, Figures S1 and S2, and Tables S1, S5, and S6 [file mmc1.pdf]

Stem Cell Reports, Volume *9*

Supplemental Information

Genes Associated with Pancreas Development and Function Maintain Open Chromatin in iPSCs Generated from Human Pancreatic Beta Cells

Matthias Thurner, Liraz Shenhav, Agata Wesolowska-Andersen, Amanda J. Bennett, Amy Barrett, Anna L. Gloyn, Mark I. McCarthy, Nicola L. Beer, and Shimon Efrat

Tissue	Donor ID	Sample ID	Passage #	Karyotype	ATAC-seq	RNA-seq
Fibroblast derived	Individual A	FiPSC_A.1	24-25	46XX	✓	DE+PP stage
		FiPSC_A.2	24-26	46XX	✓	DE+PP stage
		FiPSC_A.3	21	46XX	✓	NA
	Individual B	FiPSC_B.1	20-26	46XY	✓	DE+PP stage
		FiPSC_B.2	18-24	46XY	✓	DE+PP stage
Beta-cell derived	Individual C	BiPSC_C.1	11-12	46XX	✓	DE+PP stage
		BiPSC_C.2	10-12	46XX	✓	DE+PP stage
	Individual D	BiPSC_D.1	9-10	47XY+20	✓	DE+PP stage
		BiPSC_D.2	10	47XY+20	✓	NA
	Individual E	BiPSC_E.1	9-11	46XX	✓	DE+PP stage

Table S1, related to Figure 1. Sample characteristics. RNA-seq data for each stage was collected in duplicates. Abbreviations: Definitive Endoderm (DE); Pancreatic Progenitor (PP)

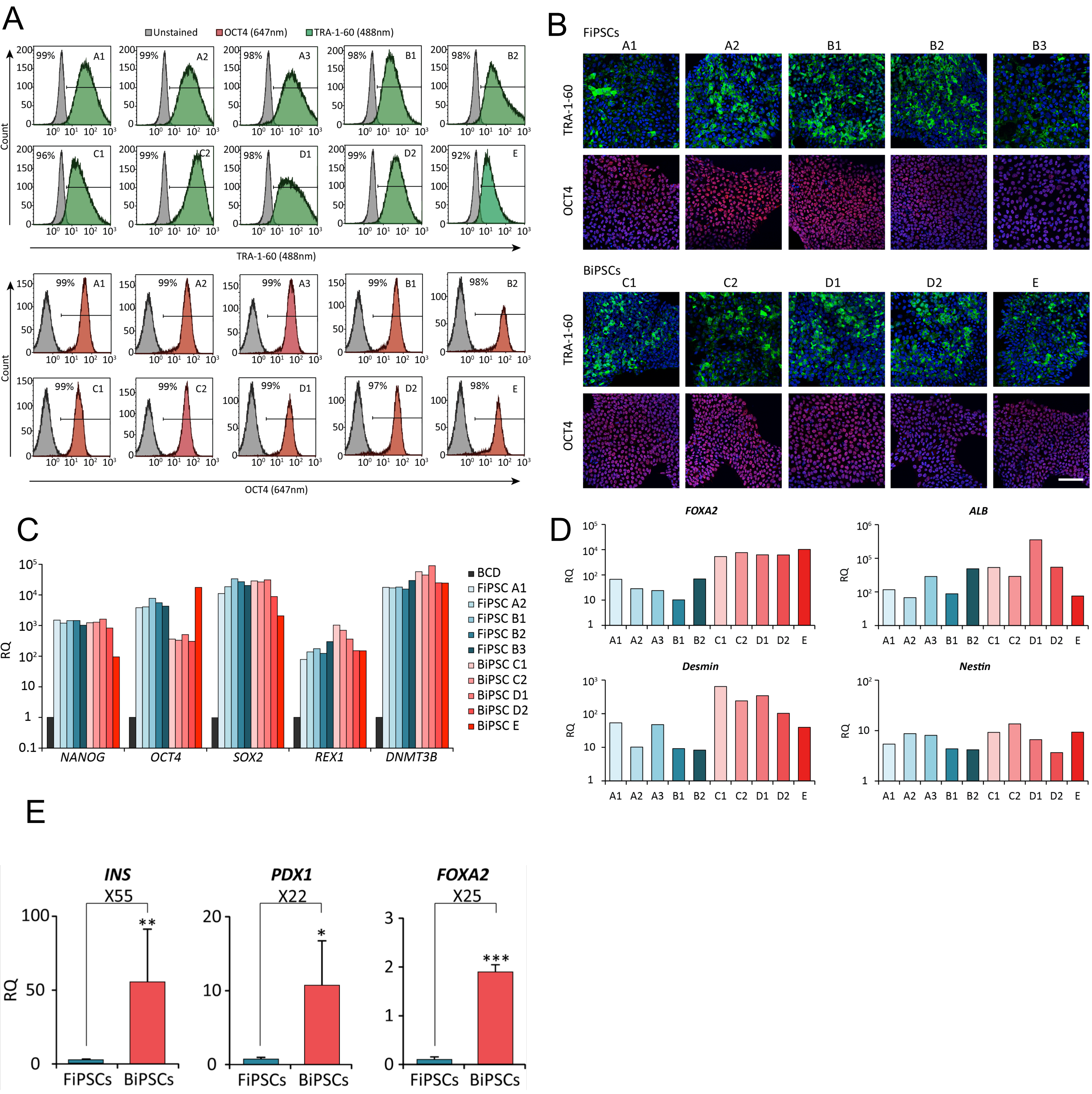

Figure S1, related to Figure 1. iPSC lines characterisation. **A:** FACS analysis of TRA-1-60 and OCT4 in FiPSCs (A1-B2) and BiPSCs (C1-E) clones. **B** Immunofluorescence analysis of TRA-1-60 and OCT4 in iPSC clones. Scale bar=100µm. **C:** qPCR analysis of pluripotency gene expression in iPSC lines in comparison to BCDs (RQ=1). Results were normalized to GAPDH. **D**: qPCR analysis of representative markers from the three embryonic germ layers in EBs differentiated from BiPSCs. FOXA2 (endoderm), ALB (endoderm), Desmin (mesoderm) and Nestin (ectoderm). Values are compared to undifferentiated hESC-H1 (RQ=1) and are normalized to GAPDH. **E:** qPCR analysis of *INS*, *PDX1* and *FOXA2* transcripts in 21-day EBs derived from BiPSCs (n=5) and FiPSCs (n=5), in comparison to EBs derived from hESC- H1 (RQ=1). Values are mean±SE and are normalized to GAPDH. *P- value<0.05; **P-value-<0.001, ***P- value-<0.0001.

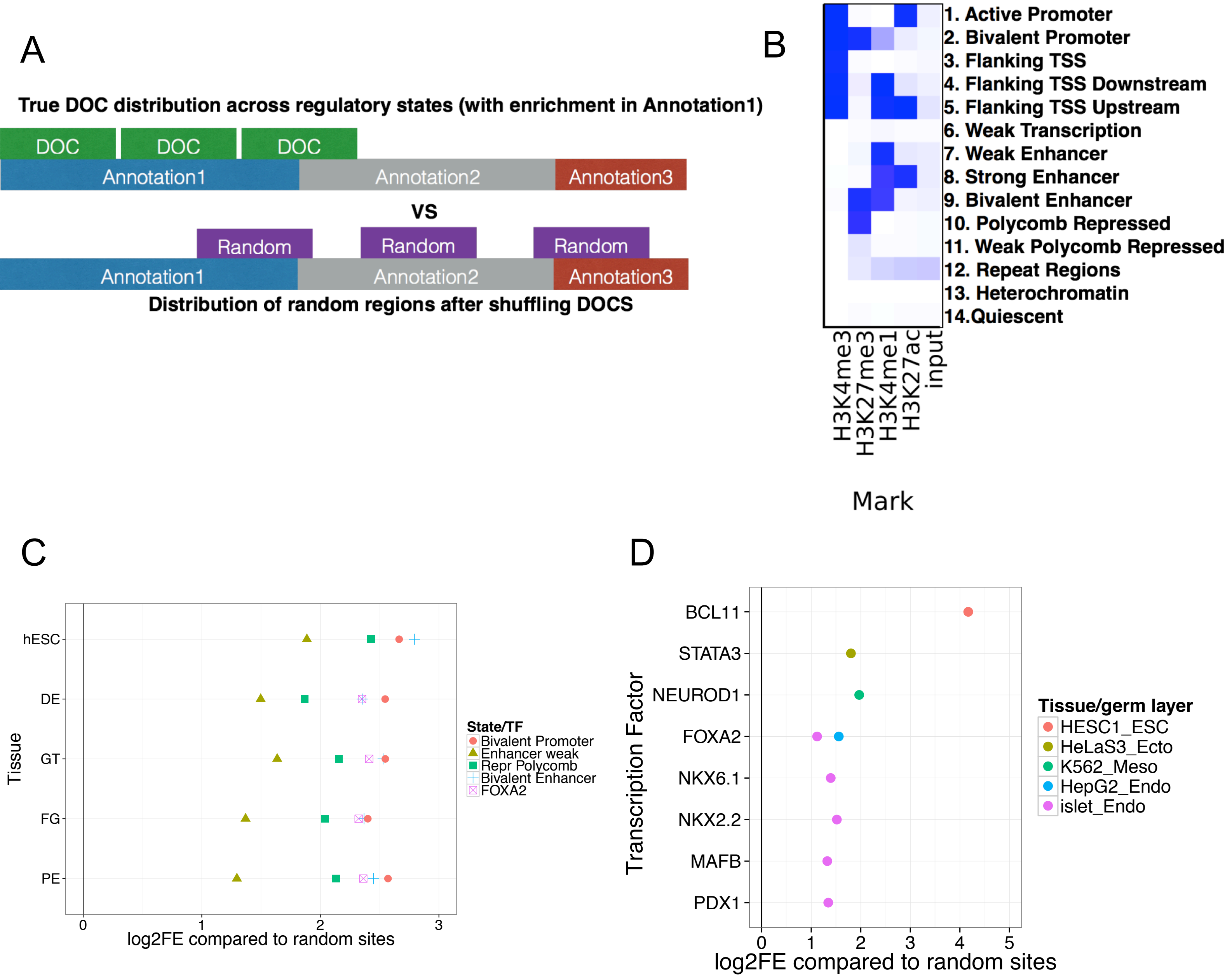

Figure S2, related to Figure 2. DOCS enrichment across various regulatory annotations compared to random sites. A) Schematic diagram of evaluating enrichment of DOCS in regulatory annotations compared to randomly shuffled regions. B) 14 State ChromHMM model predicted across stages of endodermal development using the indicated histone marks derived from Wang et al 2015 and Xie et al 2013. The colour is based on the chromHMM emission parameters and a darker colour indicates a higher frequency of a mark at a given state. C) Log2FE of DOCS (x-axis) in chromatin states and FOXA2 TFBS (indicated by shape and colour) across stages of endodermal development (y-axis). hESC (Human Stem cell, not available for FOXA2), Definitive Endoderm (DE), Gut Tube (GT), Fore Gut (FG) and Pancreatic Endoderm (PE). D) Log2FE of DOCS (x-axis) in HESC-H1 (stem cell), HeLaS3 (ectoderm), K562 (mesoderm), HepG2 (endoderm) and islet (endoderm) TFBS (y-axis) compared to random sites. Colours indicate different cell types.

Lineage tracing primers		
	Fw	Rev
**loxp's flanked**	ATCCACGCTGTTTTGACCTC	AAGTCGTGCTGCTTCATGTG

Primer sets for RNA qPCR		
	Fw	Rev
**OCT4**	AGTTTGTGCCAGGGTTTTTG	ACTTCACCTTCCCTCCAACC
**SOX2**	GGGAAATGGGAGGGGTGCAAAAGAGG	TTGCGTGAGTGTGGATGGGATTGGTG
**NANOG**	TTTGGAAGCTGCTGGGGAAG	GATGGGAGGAGGGGAGAGGA
**DNMT3B**	ATAAGTCGAAGGTGCGTCGT	GGCAACATCTGAAGCCATTT
**REX1**	TCGCTGAGCTGAAACAAATG	CCCTTCTTGAAGGTTTACAC
**GAPDH**	GTGGACCTGACCTGCCGTCT	GGAGGAGTGGGTGTCGCTGT
**PDX1**	Taqman probe Hs_00426216_m1	Taqman probe Hs_00426216_m1
**INS**	Taqman probe Hs 00355773 m1	Taqman probe Hs 00355773 m1
**FOXA2**	Taqman probe Hs_00232764_m1	Taqman probe Hs_00232764_m1
**AFP**	Taqman probe Hs_00173490_m1	Taqman probe Hs_00173490_m1
**ALB**	Taqman probe Hs_00609403_m1	Taqman probe Hs_00609403_m1
**DES**	Taqman probe Hs_00157258_m1	Taqman probe Hs_00157258_m1
**GAPDH**	Taqman probe Hs_99999905_m1	Taqman probe Hs_99999905_m1

Primer sets for ChIP-qPCR		
	Fw	Rev
**FOXA2**	TTCTTCGCTCTCAGTGCTCA	TCCGGGTCTGAACTGTAACA
**NKX2-2**	GACGACATTAACGCTGGGAC	GTCTCCTTGGAGTGGCAGAT
**INS**	GCCAGAGGAGAGAGGATCAG	TATGCAAGTCCAACGCACTG
**PDX1**	GTGGGTTCCCTCTGAGATCA	GTCCTTGTAAAGCTGCGTGG
**APRT**	GCCTTGACTCGCACTTTTGT	TAGGCGCCATCGATTTTAAG
**TEX15**	TACATTGCCCTGCCATGGTA	GCCTCCTCCTCAGAAGTTGT
**CRYAA**	TCCACCATCAGCCCCTACTA	GCCACGTCTTACCTCAGAGA

Table S5, related to Experimental Procedures. Primer sets used in the analysis. Primer sets, which were used for lineage tracing (top) and qPCR for both RNA (middle) and ChIP (bottom), are shown.

Stage	Gene direction of effect	BiDOCS	FiDOCS	DE FOXA2 targets
DE	Up in BiPSC-derived cells	**3.4E-07**	0.63	**1.4E-53**
DE	Up in FiPSC-derived cells	0.09	**2.5E-14**	1.0
PP	Up in BiPSC-derived cells	**9.1E-04**	0.96	**1.6E-03**
PP	Up in FiPSC-derived cells	**0.02**	**5.0E-08**	1.0

Table S6, related to Figure 3. Enrichment of DE and PE differentially expressed genes (hypergeometric FDR values) in Bi-DOCS and Fi-DOCS associated genes as well as active DE FOXA2 target genes. Significant enrichment (FDR<0.05) is highlighted in bold.

2. Supplemental Experimental Procedures

2.1 iPSC generation and characterization

Human pancreatic islets were received 2-4 days following isolation, dissociated into single cells, labelled for beta-cell lineage tracing and cultured as described (Russ et al., 2008). Labelled Beta-Cell-Derived (BCD) cells were sorted using a FACS Aria cell sorter (Becton Dickinson, San Jose, CA). BiPSCs were generated as previously described (Bar-Nur et al., 2011) with the following modification: sorted BCD cells were expanded to passage 8-16 and transduced with a polycistronic lentiviral vector containing the four reprogramming transcription factors OCT4, SOX2, KLF4 and c-MYC (a gift of Gustavo Mostoslavsky). ES-like colonies emerged 3-7 weeks following transduction. Five of these colonies, originating from three different human donors, were isolated and expanded for further analysis. The beta-cell origin of iPSC clones (BiPSCs) was validated by DNA PCR analysis of the recombined reporter cassette as previously described (Bar-Nur et al., 2011). BiPSC pluripotency was confirmed via FACS staining for TRA-1-60 (Biolegend, 330614) and OCT4 (BD Biosciences, 560329) using a GalliosTM Flow Cytometer (Beckman Coulter Life Sciences; 20,000–50,000 events recorded per sample).

iPSCs reprogrammed from human fibroblasts (FiPSCs) were obtained through the IMI/EU sponsored StemBANCC consortium via the Human Biomaterials Resource Centre, University of Birmingham and were generated as described previously (van de Bunt et al., 2016).

H1-hESC line was a gift of Joseph Itskovitz-Eldor. The StemBANCC consortium did not fund work associated with the H1-hESC line and the cell line was only used for comparison of the qPCR RNA expression analysis described below. FiPSC pluripotency was confirmed via FACS as before. Karyotypic QC involved: for BiPSCs G-banding as described (Bar-Nur et al., 2011), or for FiPSCs Illumina Human CytoSNP-12v2.1 beadchips (Illumina, WG-320).
iPSCs and H1-hESC cells were cultured on Matrigel™ hESC-qualified Matrix (BD Biosciences 354277) with mTeSR™1 medium (Stem Cell Technologies 05850). Embryoid bodies were generated as described previously (Bar-Nur et al., 2011). Total RNA was extracted using ZR RNA MiniPrep Kit and treated with DNase according to the manufacturer's instruction (Zymo Research, R1065). cDNA was produced by reverse transcription using High-Capacity cDNA RT Kit (Applied Biosystems, 4368813) or qScript cDNA synthesis kit (Quanta, 95047). qPCR was carried out in a 7300 real-time PCR instrument (Applied Biosystems), using FAST SYBR Green or TaqMan Universal PCR Master Mix (Applied Biosystems) with primers listed in Table S5.

Ethics statement

This study was conducted according to the principles expressed in the Declaration of Helsinki. The Institutional Review Boards of the following medical centres, which provided human islets, each provided approval for the collection of samples and subsequent analysis: University of Geneva School of Medicine; University of Minnesota; Invention Institute California. All donors provided written informed consent for the collection of all samples and subsequent analysis.
Skin fibroblast samples for reprogramming were collected with full informed consent. Ethical approval for the StemBANCC study (UK) was received from the National Research Ethics Service South Central Hampshire A research ethics committee (REC 13/SC/0179).

2.2 Human islets (from Oxford) used for ATAC-seq

Human islets were freshly isolated from cadaveric donors at the Oxford Centre for Islet Transplantation as described previously (Cross et al., 2012; van de Bunt et al., 2015) and processed for ATAC-seq (Buenrostro et al., 2013) after 1-3 days of storage in either UW (Belzer) or CMRL (without glutamine from Life Technologies) media.

Ethics Statement for the Oxford human islets

Human islet studies were approved by the Human Research Ethics Board the University of Oxford's Oxford Tropical Research Ethics Committee (OxTREC Reference: 2–15)

2.3 ATAC-seq

ATAC-seq was performed as described previously (Buenrostro et al., 2013) using ~50,000 cells per sample. Briefly, samples were lysed and transposed using T5 transposases (Illumina, FC-121-1030) and PCR (New England Labs Cat, M0541) amplified for 11 cycles to generate sequencing libraries. Libraries were purified using the Quiagen Minelute kit (28004) and Beckman Coulter Agencourt

AMPure beads (A63880) to remove primer dimers. The resulting libraries were multiplexed using primers Ad_1-6 (described in Buenrostro et al., 2013) and sequenced across 1-2 lanes of Hiseq2500 (Illumina).

2.4 Mapping and filtering of ATAC-seq reads

Raw FASTQ reads were processed with an in-house pipeline first described in (Hay et al., 2016) and on the following website http://userweb.molbiol.ox.ac.uk/public/telenius/PipeSite.html. Specifically, library and sequencing quality was checked with FASTQC (http://www.bioinformatics.babraham.ac.uk/projects/fastqc/) and reads were mapped to the human genome hg19 via bowtie version 1.1.0 (Langmead et al., 2009) with default settings but -m 2, and maxins 2000. For reads that could not be aligned the first time, adapters were removed with Trim Galore (http://www.bioinformatics.babraham.ac.uk/projects/trim_galore/) at the 3 prime end (settings -length 10, -qualFilter 20) to enhance the chance of mapping. The resulting trimmed reads were then mapped again with bowtie. Any remaining unmapped and trimmed reads were processed with FLASH version 1.2.8 settings -m 9 -x 0.125 (Magoc and Salzberg, 2011). Since overlapping paired-end reads can often not be mapped by alignment software, FLASH combines the read pair and reconstructs a read pair without overlap. These are then realigned a third time using bowtie. PCR duplicates are then removed from the mapped bam files using samtools rmdup function (Li et al., 2009). All reads overlapping any of the "unmappable" UCSC Duke blacklisted hg19 regions (https://sites.google.com/site/anshulkundaje/projects/blacklists) were also removed from the final bam file.

2.5 Peak calling and normalisation of reads

Peaks were called from filtered bam files using MACS2 (settings FDR <0.01, –nomodel, -g hs, (Zhang et al., 2008)), so as to identify regions of open chromatin. Peaks were merged for each cell type separately (human pancreatic islets, FiPSCs and BiPSC) using bedtools (Quinlan and Hall, 2010) mergeBed function. Read depth across peaks was normalised by applying the bamnormalise and bamsummary function of the software package deeptools (Ramirez et al., 2016) using standard settings.

2.6 Identification of DOCS and genomic annotations

Identification of DOCS was performed using 1000bp sliding window approach with 100bp step-size and a negative binomial test as implemented in the perl software diffReps (Shen et al., 2013). After analysing the whole genome, overlapping windows with a significant difference in normalised read depth (predefined as P<0.0001) were merged together, and then retested. Significant DOCS with an absolute minimum log2FC of 0.5 were identified after performing multiple testing correction using an FDR (FDR<0.05) approach, this based on the number of merged and retested regions. Standard settings were used apart from a defined fragment size of 50bp.

2.7 Using Epigenome Roadmap states and other epigenomic annotations to interrogate the role of DOCS

We obtained Expanded 18-state chromatin states from the Epigenome Roadmap data repository (http://egg2.wustl.edu/roadmap/web_portal/chr_state_learning.html#exp_18state). We permuted DOCS by shuffling them 1000 times using bedtools shuffleBed function, this distributing DOCS randomly across the genome. For the 98 Epigenome chromHMM states, DOCS enrichment was calculated in the following way: Each individual DOC site has an associated log2FC value indicating the difference in read depth between BiPSCs and FiPSCs. We estimated enrichment by determining the sum of log2FC for each type of chromatin state based on all true DOCS. This true value of each chromatin state was then compared to the mean of the sum of log2FC for each chromatin state derived from 1000 permutations of shuffled DOCS (true DOCS were shuffled using the shuffleBed function implemented in bedtools). For each state and each of the 98 cell types, P-values were calculated based on how often the shuffled sum of log2FC was higher compared to the true sum of log2FC (minimum P-value 0.001). The average P-value for each of the 6 germ layer/pluripotent cell types (Ectoderm, Endoderm, Mesoderm, ESC, iPSC and Others) was calculated across all cells of a given type and chromatin state. These averaged P-values were FDR corrected and used for the analysis.

We also obtained information for ChIP-seq marks from a previously-published model of endocrine pancreas development (Wang et al., 2015; Xie et al., 2013), including: H3K4me3, H3K27ac, H3K4me1, H3K27me3, Input control and FOXA2. Specifically, this model included cells that represented the following islet developmental stages: human stem cells (HSC/iPSC), Definitive

Endoderm (DE), Primitive Gut Tube (PGT), Posterior ForeGut (PFG), and Pancreatic Endoderm (PE). After aligning the FASTQ reads to the genome and filtering duplicated reads using picard tools (v1.119, http://broadinstitute.github.io/picard/), we used this histone mark information to identify chromatin regulatory states via chromHMM as described previously (Ernst and Kellis, 2012). Specifically, we identified 14 chromatin regulatory states, including bivalent enhancers (H3K27me3 and H3K4me1) and promoters (H3K4me3 and H3K27me3), weak enhancers (H3Kme1) and polycomb repressed (H3K27me3) regions. FOXA2 TFBS across these stages were also predicted using MACS2 with standard settings (apart from FDR<0.01 and including input control).

We also obtained Encode ChIP-seq transcription factor data from four Encode cell lines (EncodeProjectConsortium, 2012) representing cells derived from different germ layers. Specifically, these data represented stem cells (HESC1, TF=BCL11A Encode Experiment: ENCSR000BIP), endodermal (HepG2 cell line which is derived from a patient with liver carcinoma, TF=FOXA2, Encode Experiment: ENCSR000BNI), mesodermal (K562 cell line which is an immortalised cell line produced from a patient with chronic myelogenous leukemia, TF=NEUROD1, Encode Experiment: ENCSR986CDX) or ectodermal (HeLaS3 cell line which represents an immortalised cell line derived from a cervical cancer patient, TF=STATA3 Encode Experiment: ENCSR000EDC) lineage committment. We also made use of publicly available human islet TFBS data (FOXA2, MAFB, NKX2-2, NKX6-1, PDX1) from (Pasquali et al., 2014). FASTQ reads were processed and TFBS predicted based on MASC2 open chromatin peaks which were identified as described above.

To determine annotation enrichment, DOCS were again permuted (shuffled) 1000 times using bedtools to distribute them randomly across the genome. Enrichment was calculated by comparing the number of true DOCS overlapping a given annotation compared to the number of random DOCS overlapping the same annotation.

2.8 GREAT enrichment analysis

Bi-DOCS were tested for gene and pathway enrichment using GREAT (McLean et al., 2010) with standard settings. Pathways and GO terms with a minimum binomial and gene enrichment of 2 and an FDR < 0.05 were considered significant and reported with the associated genes. The enrichment of Bi-DOCS overlapping hESC (H1 cell line from Encode) chromatin states (including bivalent enhancer and bivalent promoter states) was determined by defining the foreground set as chromatin states (of a given type) overlapping Bi-DOCS while as background set all chromatin states (of a given type) were used. Enrichment was determined by comparing the foreground set to the background set using a hypergeometric test. As before, Pathways and GO terms with a minimum enrichment>2 and FDR<0.05 were determined as significant.

2.9 *In silico* validation

To assess the impact of genotype: Sample labels were randomized and mixed (3 BiPSCs and 2 FiPSCs versus 3 FiPSCs and 2 BiPSCs). The newly generated randomised sample sets were reanalysed using diffReps as described above so as to evaluate tissue of origin and genotype effects by comparing the number of true VS random DOCS.

To assess the impact of karyotype: samples with abnormal karyotypes were removed and the diffReps analysis repeated as before. Correlation between DOCS identified in all samples and DOCS identified in karyotypically normal samples was evaluated using R.

Enrichment of BiDOCS in stage endodermal regulatory genes: publicly available stage specific endodermal regulatory genes were obtained from (van de Bunt et al., 2016). Bi-DOCS regions and islet ATAC-seq open chromatin regions were linked to genes through GREAT (McLean et al., 2010) and enrichment of Bi-DOCS associated genes (versus islet open chromatin associated genes) in stage specific endodermal regulatory genes was determined using a fisher test.

2.10 ChIP-seq qPCR validation

Cells were treated with 1% formaldehyde for 10 minutes at room temperature before lysis in buffer containing: 10%SDS, 10mM EDTA pH8, and 50mM Tris-HCl pH 8.1 (10 minutes at 4 ^{O}C). Chromatin was sonicated to 200-500-bp fragments using Bioruptor® Plus sonication device (Diagenode). Sonicated DNA fragment sizes range was validated by agarose gel electrophoresis. Immunoprecipitation was performed overnight using anti-H3K4me3 antibody (Abcam ab8580) or normal rabbit IgG (Millipore 12-370). Crosslinking reversal was preformed overnight at 65°C with proteinase K (Thermo scientific EO0491)). DNA was recovered using a PCR clean-up kit (Qiagen 28004). Eluted DNA fragments were used for qPCR analysis with primers listed in Table S4.

Enrichment of open chromatin was validated using primers recognizing the APRT promoter (data not shown). Normal rabbit IgG was used to determine background levels.

2.11 iPSC differentiation

Four FiPSCs lines (from two human donors) and four BiPSCs lines (from three human donors) were induced to differentiate into definitive endoderm (DE) and pancreatic progenitors (PP), using a published protocol (Rezania et al., 2014). These eight iPSCs lines were the same lines used for the ATAC-seq analysis, and in the same range of passage number (Table S1). The differentiation was carried out in duplicates. RNA was collected at the end of each differentiation stage for RNA-seq analysis.

2.12 RNA-seq data processing and analysis

The TruSeq stranded paired-end RNA-seq libraries were prepared for a total of 32 samples and sequenced across 2 lanes of Illumina HiSeq2500 to a mean depth of 36.3 millions (±4.3 million) raw sequencing read pairs per sample. The sequencing quality was assessed with FASTQC (http://www.bioinformatics.babraham.ac.uk/projects/fastqc/) and the raw sequencing reads were mapped to the human genome hg19 using STAR version 2.5.1 (Dobin et al., 2013) with default settings. The GENCODE v19 GTF was applied to guide the spliced alignment. Duplicated alignments were marked with the MarkDuplicates script from the Picard tools v2.1.1 suite (http://broadinstitute.github.io/picard). Gene expression was quantified with featureCounts (Liao et al., 2014) using the GENCODE v19 GTF file. We restricted the downstream analysis to only the annotated protein-coding genes. Differential expression analysis between BiPSC and FiPSC at each differentiation stage was performed using DESeq2 R package (package v.1.10.1, under R v.3.2.5) (Love et al., 2014) using 5% FDR as the cut-off for reporting of the statistically significant results.

2.13 Differentially expressed gene list enrichment analysis

As previously described, we used GREAT (McLean et al., 2010) to assign genes to Bi-DOCS and Fi-DOCS regions, as well as the previously reported stage specific FOXA2 ChIP-seq peaks (Wang et al., 2015) at the definitive endoderm (DE) differentiation stage. We restricted the investigated FOXA2 targets to genes expressed at >1TPM in all samples at the DE stage. The significance of enrichment of genes upregulated in each lineage at the DE and PP stages within the Bi-DOCS, Fi-DOCS and FOXA2 targets genes lists was calculated using the upper tail of the hypergeometic distribution and the resulting p-values were adjusted for multiple testing using the Benjamini & Hochberg method of the p.adjust function within R v.3.2.5.

2.14 Statistical analysis and graphical visualisation

Statistical analysis (PCs, Spearman's rho and hierarchical clustering) and graphical visualisation was performed using R (version 3.0.2 (R-Core-Team, 2013)) and the ggplot2 package (Wickham, 2009).

2.15 Data access

Data has been deposited at the EBI hosted European Genome-phenome Archive (EGA, http://www.ebi.ac.uk/ega/) and European Nucleotide Archive (ENA, http://www.ebi.ac.uk/ena). EGA accession: EGAS00001002591 (islet ATAC-seq and iPSC RNA-seq). ENA accession: PRJEB21856 (iPSC ATAC-seq).

3. Supplemental References:

Buenrostro, J.D., Giresi, P.G., Zaba, L.C., Chang, H.Y., and Greenleaf, W.J. (2013). Transposition of native chromatin for fast and sensitive epigenomic profiling of open chromatin, DNA-binding proteins and nucleosome position. Nat Methods *10*, 1213-1218.

Cross, S.E., Hughes, S.J., Clark, A., Gray, D.W., and Johnson, P.R. (2012). Collagenase does not persist in human islets following isolation. Cell Transplant *21*, 2531-2535.

Dobin, A., Davis, C.A., Schlesinger, F., Drenkow, J., Zaleski, C., Jha, S., Batut, P., Chaisson, M., and Gingeras, T.R. (2013). STAR: ultrafast universal RNA-seq aligner. Bioinformatics *29*, 15-21.

Ernst, J., and Kellis, M. (2012). ChromHMM: automating chromatin-state discovery and characterization. Nat Methods *9*, 215-216.

Hay, D., Hughes, J.R., Babbs, C., Davies, J.O., Graham, B.J., Hanssen, L.L., Kassouf, M.T., Oudelaar, A.M., Sharpe, J.A., Suciu, M.C*., et al.* (2016). Genetic dissection of the alpha-globin super-enhancer in vivo. Nat Genet *48*, 895-903.

Langmead, B., Trapnell, C., Pop, M., and Salzberg, S.L. (2009). Ultrafast and memory-efficient alignment of short DNA sequences to the human genome. Genome Biol *10*, R25.

Li, H., Handsaker, B., Wysoker, A., Fennell, T., Ruan, J., Homer, N., Marth, G., Abecasis, G., Durbin, R., and Genome Project Data Processing, S. (2009). The Sequence Alignment/Map format and SAMtools. Bioinformatics *25*, 2078-2079.

Liao, Y., Smyth, G.K., and Shi, W. (2014). featureCounts: an efficient general purpose program for assigning sequence reads to genomic features. Bioinformatics *30*, 923-930.

Love, M.I., Huber, W., and Anders, S. (2014). Moderated estimation of fold change and dispersion for RNA-seq data with DESeq2. Genome Biol *15*, 550.

Magoc, T., and Salzberg, S.L. (2011). FLASH: fast length adjustment of short reads to improve genome assemblies. Bioinformatics *27*, 2957-2963.

McLean, C.Y., Bristor, D., Hiller, M., Clarke, S.L., Schaar, B.T., Lowe, C.B., Wenger, A.M., and Bejerano, G. (2010). GREAT improves functional interpretation of cis-regulatory regions. Nat Biotechnol *28*, 495-501.

Quinlan, A.R., and Hall, I.M. (2010). BEDTools: a flexible suite of utilities for comparing genomic features. Bioinformatics *26*, 841-842.

R-Core-Team (2013). R: A Language and Environment for Statistical Computing (VIenna, Austria: R Foundation for Statistical Computing).

Ramirez, F., Ryan, D.P., Gruning, B., Bhardwaj, V., Kilpert, F., Richter, A.S., Heyne, S., Dundar, F., and Manke, T. (2016). deepTools2: a next generation web server for deep-sequencing data analysis. Nucleic Acids Res *44*, W160-165.

Russ, H.A., Bar, Y., Ravassard, P., and Efrat, S. (2008). In vitro proliferation of cells derived from adult human beta-cells revealed by cell-lineage tracing. Diabetes *57*, 1575-1583.

Shen, L., Shao, N.Y., Liu, X., Maze, I., Feng, J., and Nestler, E.J. (2013). diffReps: detecting differential chromatin modification sites from ChIP-seq data with biological replicates. PLoS One *8*, e65598.

van de Bunt, M., Manning Fox, J.E., Dai, X., Barrett, A., Grey, C., Li, L., Bennett, A.J., Johnson, P.R., Rajotte, R.V., Gaulton, K.J*., et al.* (2015). Transcript Expression Data from Human Islets Links Regulatory Signals from Genome-Wide Association Studies for Type 2 Diabetes and Glycemic Traits to Their Downstream Effectors. PLoS genetics *11*, e1005694.

Wickham, H. (2009). ggplot2: Elegant Graphics for Data Analysis (New York: Springer-Verlag).

Zhang, Y., Liu, T., Meyer, C.A., Eeckhoute, J., Johnson, D.S., Bernstein, B.E., Nusbaum, C., Myers, R.M., Brown, M., Li, W*., et al.* (2008). Model-based analysis of ChIP-Seq (MACS). Genome Biol *9*, R137.
