# Supplementary material for: Genes Associated with Pancreas Development and Function Maintain Open Chromatin in iPSCs Generated from Human Pancreatic Beta Cells
Source: Stem Cell Reports. 2017 Nov 1;9(5):1395–405. doi: 10.1016/j.stemcr.2017.09.020 (PMC5831005; doi:10.1016/j.stemcr.2017.09.020)
Supplement: Document S2. Article plus Supplemental Information [file mmc5.pdf]

Stem Cell Reports
Report

ISSCR
OPEN ACCESS

Genes Associated with Pancreas Development and Function Maintain Open Chromatin in iPSCs Generated from Human Pancreatic Beta Cells

Matthias Thurner,[1,2,5] Liraz Shenhav,[3,5] Agata Wesolowska-Andersen,[1] Amanda J. Bennett,[2] Amy Barrett,[2] Anna L. Gloyn,[1,2,4] Mark I. McCarthy,[1,2,4] Nicola L. Beer,[2,*] and Shimon Efrat[3]
[1]The Wellcome Trust Centre for Human Genetics, University of Oxford, Oxford, UK
[2]Oxford Centre for Diabetes, Endocrinology and Metabolism, University of Oxford, Oxford, UK
[3]Department of Human Molecular Genetics and Biochemistry, Sackler School of Medicine, Tel Aviv University, Tel Aviv, Israel
[4]Oxford NIHR Biomedical Research Centre, Churchill Hospital, Oxford, UK
[5]Co-first author
*Correspondence: nicolabeer.nb@gmail.com
https://doi.org/10.1016/j.stemcr.2017.09.020

SUMMARY

Current *in vitro* islet differentiation protocols suffer from heterogeneity and low efficiency. Induced pluripotent stem cells (iPSCs) derived from pancreatic beta cells (BiPSCs) preferentially differentiate toward endocrine pancreas-like cells versus those from fibroblasts (FiPSCs). We interrogated genome-wide open chromatin in BiPSCs and FiPSCs via ATAC-seq and identified ~8.3k significant, differential open chromatin sites (DOCS) between the two iPSC subtypes (false discovery rate [FDR] < 0.05). DOCS where chromatin was more accessible in BiPSCs (Bi-DOCS) were significantly enriched for known regulators of endodermal development, including bivalent and weak enhancers, and FOXA2 binding sites (FDR < 0.05). Bi-DOCS were associated with genes related to pancreas development and beta-cell function, including transcription factors mutated in monogenic diabetes (*PDX1*, *NKX2-2*, *HNF1A*; FDR < 0.05). Moreover, Bi-DOCS correlated with enhanced gene expression in BiPSC-derived definitive endoderm and pancreatic progenitor cells. Bi-DOCS therefore highlight genes and pathways governing islet-lineage commitment, which can be exploited for differentiation protocol optimization, diabetes disease modeling, and therapeutic purposes.

INTRODUCTION

Human pancreatic islets have been placed center stage in type 2 diabetes pathogenesis (Dimas et al., 2014). Current disease-modeling efforts are often frustrated by the limited availability of human physiologically authentic islet-like cells. Derivation of endocrine pancreas from iPSCs represents one solution for generating sufficient numbers of physiologically and disease-relevant human islet-like cells (Nostro et al., 2015; Pagliuca et al., 2014; Rezania et al., 2014). While directed *in vitro* differentiation of iPSCs routinely yields cells positive for islet hormones, such as insulin and glucagon, these cell populations are heterogeneous, contain poly-hormonal cells, and are functionally immature versus primary human islets (Rezania et al., 2014; van de Bunt et al., 2016). Differentiation efficiency also varies across iPSC lines (Bar-Nur et al., 2011; Burrows et al., 2016; Kim et al., 2010; Kyttala et al., 2016; Polo et al., 2010; Rouhani et al., 2014).

Possible causes for inconsistencies in differentiation capacity include technical factors such as reprogramming strategy (Balboa and Otonkoski, 2015), but also line-specific characteristics such as donor genotype (Burrows et al., 2016; Kyttala et al., 2016; Rouhani et al., 2014). There is also evidence to support an epigenetic "memory" in iPSCs (Bar-Nur et al., 2011; Kim et al., 2010; Polo et al., 2010), this comprising epigenomic and transcriptomic signatures of the original reprogrammed cell type, which may erode over prolonged periods of passaging in culture (Bar-Nur et al., 2011; Kim et al., 2010; Polo et al., 2010).

The epigenome plays an important role in establishing developmental competence in stem cells (Wang et al., 2015; Xie et al., 2013). Epigenetic memory could thus account for the enhanced propensity of beta-cell-derived iPSCs (BiPSCs) to differentiate down the endocrine pancreas lineage versus those derived from skin fibroblasts (FiPSCs; Bar-Nur et al., 2011). Here, we aimed to capitalize upon proposed BiPSC epigenetic memory to identify genes and pathways governing islet development and identity. By utilizing an assay for transposase accessible chromatin with high-throughput sequencing (ATAC-seq), we first aimed to define the open chromatin landscape in BiPSCs. Secondly, by comparison with global open chromatin in FiPSCs, we aimed to identify BiPSC-specific differential open chromatin sites (Bi-DOCS). We then integrated Bi-DOCS with publicly available genomic annotations to highlight regulatory elements, genes, and pathways that may explain preferential differentiation of BiPSCs toward endocrine pancreas-like cells.

Finally, to confirm that differences in open chromatin lead to changes in gene expression, we compared the transcriptome of cells derived from directed differentiation of both BiPSCs and FiPSCs toward two key stages of islet development (definitive endoderm and pancreatic

CrossMark

This is an open access article under the CC BY license (http://creativecommons.org/licenses/by/4.0/).

progenitors). Our study improves understanding of human islet development, which may provide clues for improving *in vitro* differentiation protocols, facilitate more advanced disease modeling, and eventually contribute to therapeutic applications (Rezania et al., 2014; ViaCyte, 2014).

RESULTS

Mapping the Open Chromatin Landscape in BiPSCs

We generated five BiPSC lines from three independent non-diabetic donors and five FiPSC lines from two independent non-diabetic donors using reprogramming strategies described previously (Bar-Nur et al., 2011; van de Bunt et al., 2016) (Table S1). Of note, because of differences in source, the BiPSCs were on average ten passages lower than the FiPSC lines. All iPSC lines passed quality control assessing pluripotency and differentiation capacity (Figures S1A–S1D). All but two lines (BiPSC-D1 and D2, Table S1) were karyotypically normal; however, as shown in the section on "*In Silico* and Cellular Validation of DOCS", removing these two lines did not substantially alter the results reported in this study. As shown previously (Bar-Nur et al., 2011), BiPSCs showed enhanced spontaneous *in vitro* differentiation capacity into islet-lineage cells expressing *FOXA2*, *PDX1*, and *INS,* compared with FiPSCs (Figure S1E).

We performed ATAC-seq in all ten lines, as well as in five adult human islet samples. We generated between 28 and 186 million mapped and filtered reads per sample (Figure 1A), and identified between 17.3k and 123.9k open chromatin peaks (false discovery rate [FDR] < 0.01). Consistent with the pluripotent nature of both iPSC subtypes, we found the open chromatin pattern to be highly similar between BiPSCs and FiPSCs (median rho = 0.84, Figure 1B), and clearly distinct from primary human islets. Principal component analysis (PCA) across open chromatin peaks of all samples also confirmed that the two iPSC types were highly similar, and that iPSCs did not cluster by donor genotype (Figure 1C).

Identifying BiPSC-Specific Open Chromatin

Despite the similarities in open chromatin between the two iPSC subtypes, we were able to identify differential open chromatin sites (DOCS) between BiPSCs and FiPSCs (Supplemental Experimental Procedures). We found 8.3k significant DOCS (FDR < 0.05) with a minimum absolute log2 fold change (log2FC) of 0.5 (Figure 1D). About 4.8k (58%) of DOCS were characterized by an increase in open chromatin in BiPSCs (Bi-DOCS), while the remaining 3.5k sites were more open in FiPSCs (Fi-DOCS). PCA and hierarchical clustering of normalized ATAC-seq read depth across DOCS showed that BiPSCs were more similar to human islets than FiPSCs (Figures 1E and 1F). Again, we did not observe sample clustering by donor. This suggests that genetic variation is not the predominant driver of differences in open chromatin in our study.

Bi-DOCS Are Enriched in Chromatin States Involved in Developmental Competence

To uncover the potential regulatory landscape of DOCS, we obtained data on 18 predefined chromatin states from 98 Epigenome Roadmap cell types, which include human islets (Kundaje et al., 2015). Samples were grouped according to pluripotency (iPSCs and embryonic stem cells [ESCs], cell types per group = 4–5), germ layer (mature cells and tissues, cell types per group = 17–42), or "other" (germ layer status unclear, cell type number = 10). We found through permutation that DOCS were significantly enriched in enhancer, promoter, flanking transcription start site (TSS) and polycomb-repressed chromatin states, compared with random regions (mean FDR adjusted p value across all cell types of a given chromatin and germ layer/stem cell type < 0.05 and log2 fold enrichment (log2FE) > 0; Figures 2A and S2A).

We observed that across groups Bi-DOCS were enriched for bivalent enhancers, bivalent promoters, weak enhancers, flanking TSS downstream elements, and polycomb-repressed regions versus Fi-DOCS (mean log2FE ratio of Bi-DOCS versus Fi-DOCS (log2FE ratio) > 0, Figure 2B). In contrast, Fi-DOCS showed significant enrichment in ESC and iPSC active enhancer chromatin states compared with Bi-DOCS (log2FE ratio < 0, Figure 2B). These results show that Bi-DOCS overlap important regulatory elements known to be involved in developmental competence, including endoderm developmental competence (weak enhancer and bivalent states; Wang et al., 2015; Xie et al., 2013).

Bi-DOCS Are Enriched for Regulators of Early Endodermal Lineage Commitment

Endoderm developmental competence is regulated by epigenetic factors, including weak enhancers (marked by H3K4me1), bivalent regions marked by H3K27me3 (bivalent enhancers/promoters), and changes in gene expression governed by lineage-specific transcription factors such as FOXA2 (Wang et al., 2015; Xie et al., 2013). To confirm whether Bi-DOCS were enriched for such early endodermal regulatory states, we identified FOXA2 transcription factor binding sites (TFBS) and chromatin regulatory states across discrete stages of a previously published model of pancreatic endoderm development (Figure S2B; Supplemental Experimental Procedures) (Wang et al., 2015; Xie et al., 2013). We found that Bi-DOCS (versus Fi-DOCS) showed significant enrichment in weak enhancers,

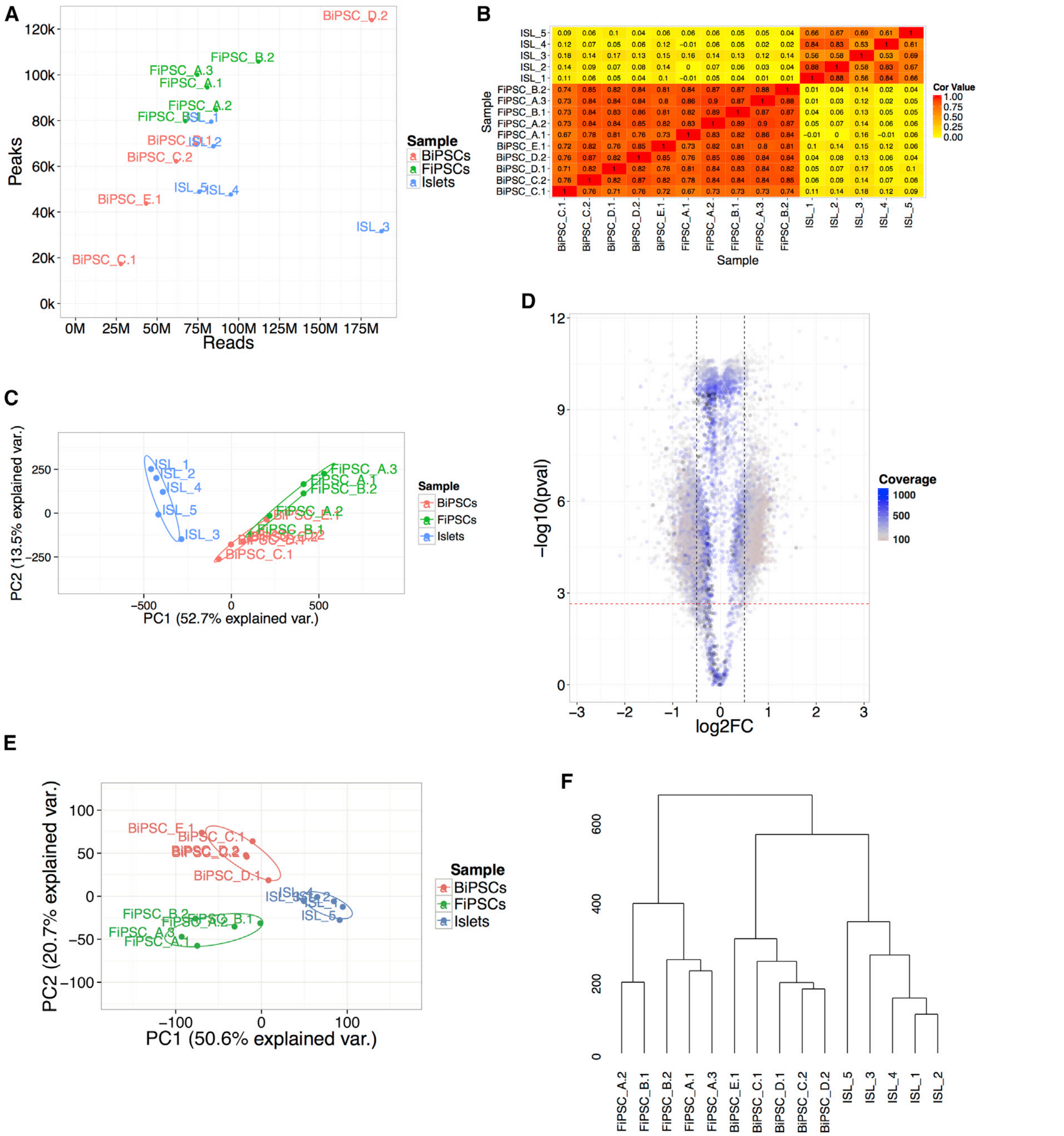

Figure 1. The Open Chromatin Landscape of BiPSCs and FiPSCs
(A) Sample read (x axis) and peak number (y axis).
(B) Heatmap with mean sample correlation (rho) across peaks.
(C) Sample PCA plot based on peak read depth.
(D) DOCS Volcano plot with log2FC (x axis) and −log10 p value (y axis). DOCS, dashed black (log2FC ± 0.5) and red (FDR < 0.05) lines.
(E) Sample PCA plot based on DOCS read depth.
(F) Hierarchical clustering of samples based on DOCS read depth.
Color of cell type in (A, C, and E), BiPSCs (red), FiPSCs (green), human islets (blue).

bivalent enhancers, bivalent promoters, polycomb-repressed regions, and FOXA2 TFBS as identified across all stages of pancreatic endoderm development (FDR < 0.05, log2FE ratio > 0, Figures 2C and S2C). Enrichment for these regulatory regions in Bi-DOCS may explain the preferential endodermal lineage commitment of BiPSCs.

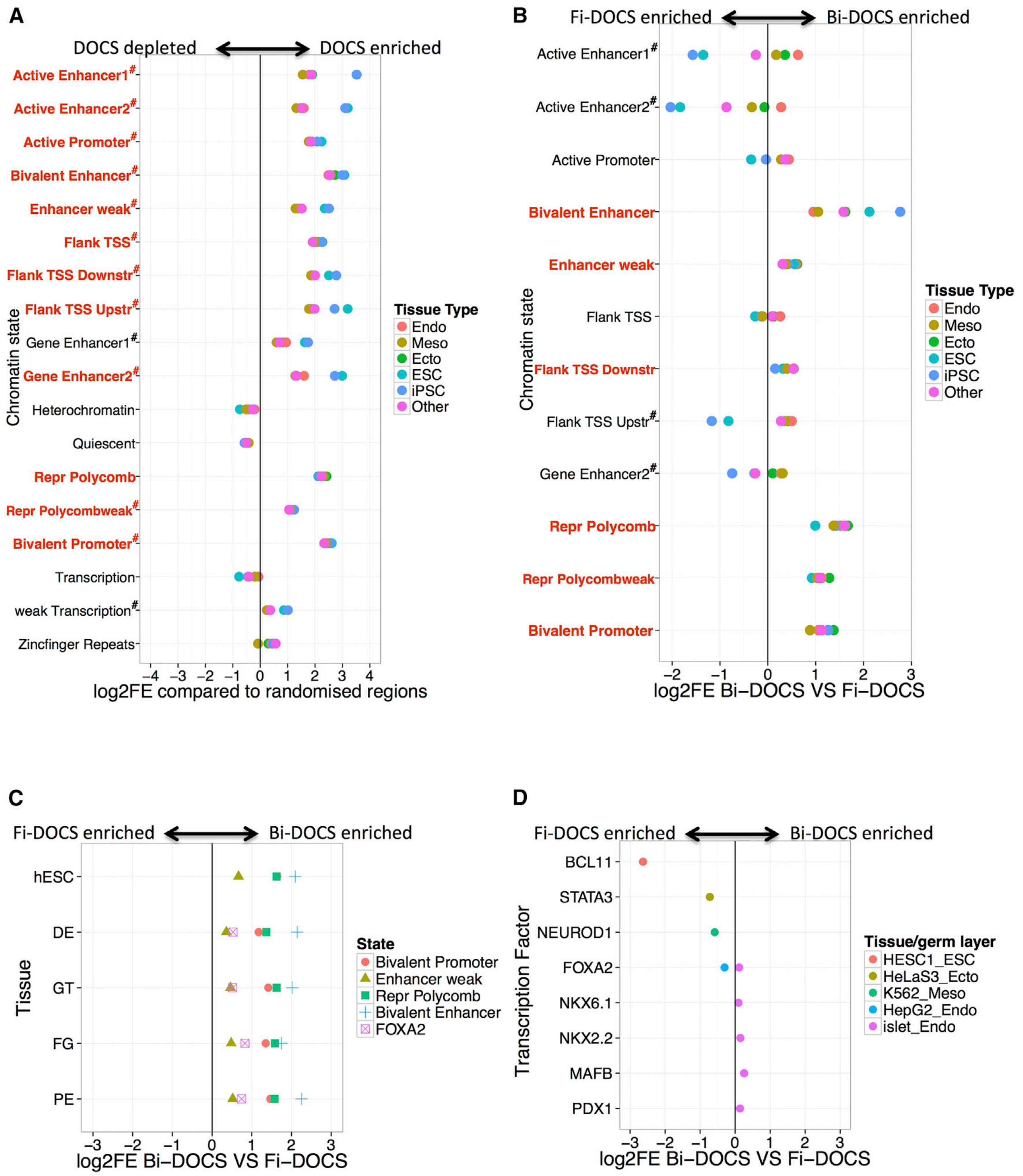

Figure 2. Enrichment of Bi-DOCS and Fi-DOCS in Chromatin States

(A) Enrichment (x axis) of DOCS in Epigenome Roadmap chromatin states (y axis) grouped according to pluripotent cell/germ layer type. Bold red states were enriched across all germ layer and pluripotent stem cell states while # indicates that ESC/iPSC states had highest enrichment.

(B) Log2FE ratio (x axis) of Bi-DOCS versus Fi-DOCS across chromatin states (y axis) enriched in (A). Bold red states are enriched for Bi-DOCS while # shows states most enriched for Fi-DOCS in ESC/iPSC lines.

(C) Log2FE ratio (x axis) of Bi-DOCS versus Fi-DOCS across chromatin states and FOXA2 TFBS (shape/color) identified during different stages (y axis) of endodermal development. human stem cells (hESC, FOXA2 not available), definitive endoderm (DE), gut tube (GT), fore gut (FG), and pancreatic endoderm (PE).

(D) Log2FE ratio (x axis) of Bi-DOCS versus Fi-DOCS across HESC-H1 (stem cell, red), HeLaS3 (ectoderm, dark yellow), K562 (mesoderm, green), HepG2 (endoderm, blue), and islet (endoderm, purple) TFBS (y axis).

Table 1. Bi-DOCS-Associated GO Biological Processes and MSigDB Pathways Terms

Term	Pathway Description	DOCS Trend	Gene Number	Region Enrichment	Gene Enrichment	Regional FDR	Gene FDR	Associated Function
GO	neural tube patterning	Bi-DOCS	80	3.27	2.69	8.7×10^{-17}	2.4×10^{-5}	neuro-developmental
GO	positive regulation of embryonic development	Bi-DOCS	51	4.75	3.23	1.0×10^{-16}	9.3×10^{-3}	developmental
GO	non-canonical Wnt receptor signaling pathway	Bi-DOCS	65	3.55	2.10	2.4×10^{-15}	3.9×10^{-2}	WNT/NOTCH signaling
GO	hindbrain morphogenesis	Bi-DOCS	82	2.84	2.32	4.1×10^{-14}	4.1×10^{-4}	neuro-developmental
GO	branching involved in mammary gland duct morphogenesis	Bi-DOCS	68	3.20	2.53	5.2×10^{-14}	3.0×10^{-3}	developmental
GO	cerebellum morphogenesis	Bi-DOCS	75	2.86	2.36	4.9×10^{-13}	6.8×10^{-4}	neuro-developmental
GO	mammary gland duct morphogenesis	Bi-DOCS	94	2.39	2.47	6.2×10^{-12}	1.8×10^{-4}	developmental
GO	mammary gland epithelium development	Bi-DOCS	123	2.10	2.12	9.1×10^{-12}	1.4×10^{-4}	developmental
GO	dorsal spinal cord development	Bi-DOCS	51	3.42	2.57	1.4×10^{-11}	3.8×10^{-3}	neuro-developmental
GO	cerebellum development	Bi-DOCS	110	2.10	2.02	1.4E-10	1.1×10^{-4}	neuro-developmental
GO	cerebellar cortex morphogenesis	Bi-DOCS	58	2.91	2.31	1.9E-10	6.6×10^{-3}	neuro-developmental
GO	spinal cord association neuron differentiation	Bi-DOCS	38	3.83	2.69	5.6E-10	1.6×10^{-2}	neuro-developmental
GO	dorsal/ventral neural tube patterning	Bi-DOCS	50	2.99	2.83	2.0×10^{-9}	9.5×10^{-4}	neuro-developmental
GO	negative regulation of Notch signaling pathway	Bi-DOCS	34	3.73	2.55	1.1×10^{-8}	1.0×10^{-2}	WNT/NOTCH signaling
GO	cell differentiation in spinal cord	Bi-DOCS	93	2.07	2.83	1.1×10^{-8}	2.6×10^{-9}	neuro-developmental
GO	cell differentiation in hindbrain	Bi-DOCS	55	2.61	2.64	2.4×10^{-8}	1.6×10^{-3}	neuro-developmental
GO	cerebellar cortex development	Bi-DOCS	65	2.38	2.02	2.8×10^{-8}	8.8×10^{-3}	neuro-developmental
GO	somitogenesis	Bi-DOCS	93	2.00	2.05	4.6×10^{-8}	4.1×10^{-4}	developmental
GO	white fat cell differentiation	Bi-DOCS	29	3.37	2.69	1.3×10^{-6}	4.5×10^{-2}	developmental
GO	embryonic axis specification	Bi-DOCS	65	2.10	2.02	2.0×10^{-6}	1.9×10^{-2}	developmental
GO	ventral spinal cord interneuron fate commitment	Bi-DOCS	34	2.91	3.67	2.8×10^{-6}	3.1×10^{-4}	neuro-developmental
GO	regulation of insulin receptor signaling pathway	Bi-DOCS	44	2.44	2.02	5.0×10^{-6}	3.3×10^{-2}	diabetes/insulin

(*Continued on next page*)

Table 1. *Continued*

Term	Pathway Description	DOCS Trend	Gene Number	Region Enrichment	Gene Enrichment	Regional FDR	Gene FDR	Associated Function
GO	positive regulation of focal adhesion assembly	Bi-DOCS	25	3.46	2.80	5.7×10^{-6}	1.9×10^{-2}	other
GO	cerebellar cortex formation	Bi-DOCS	40	2.54	2.43	6.4×10^{-6}	1.8×10^{-2}	neuro-developmental
GO	regulation of Notch signaling pathway	Bi-DOCS	58	2.06	2.21	1.4×10^{-5}	1.1×10^{-3}	WNT/NOTCH signaling
GO	spinal cord dorsal/ventral patterning	Bi-DOCS	39	2.34	2.55	4.3×10^{-5}	1.0×10^{-2}	neuro-developmental
GO	ventral spinal cord interneuron differentiation	Bi-DOCS	35	2.46	3.42	5.0×10^{-5}	4.1×10^{-4}	neuro-developmental
MSigDB pathway	Wnt signaling network	Bi-DOCS	53	2.68	2.31	9.4×10^{-8}	2.8×10^{-2}	WNT/NOTCH signaling
MSigDB pathway	maturity-onset diabetes of the young	Bi-DOCS	40	2.15	2.43	3.0×10^{-4}	2.5×10^{-2}	diabetes/insulin

Bi-DOCS Enrichment Is Specific to Pancreatic Endoderm Lineage Commitment

To show that Bi-DOCS are specifically enriched for pancreatic endoderm regulatory annotations and not regulatory annotations in other cell types or mature islets, we obtained publicly available TFBS information for four Encode cell lines (representative of pluripotency or germ layer commitment) (Encode Project Consortium, 2012) and TFBS active in human islets (Pasquali et al., 2014; Figures 2D and S2D).

While we found strong enrichment of all DOCS in all TFBS (Figure S2D), Bi-DOCS (compared with Fi-DOCS) were not enriched for TFBS from any of the four tested cell lines (Figure 2D), and showed only weak global enrichment in TFBS from adult primary human islets (max log2FE ratio = 0.3; Figures 2D and S2D).

These data confirm that Bi-DOCS are highly and specifically enriched for TFBS and chromatin states active in early pancreatic endoderm development (see previous section). However, despite this global pattern, we also found a number of Bi-DOCS associated with mature beta-cell genes including *INS* and *PDX1* as described below.

Bi-DOCS Are Enriched for Genes and Pathways Regulating Pancreatic Islet Development and Function

To understand which genes and pathways are regulated by regions mapping to Bi-DOCS, we conducted a pathway enrichment test (Supplemental Experimental Procedures). We found that the 4.8k Bi-DOCS were significantly enriched in 27 Gene Ontology (GO) Biological Process and 2 MSigDB Pathway terms (binomial and hypergeometric FDR < 0.05 and binomial region fold enrichment and minimum gene enrichment > 2; Tables 1 and S2). Stratification of Bi-DOCS into different regulatory annotations highlighted additional terms in which Bi-DOCS were significantly enriched (hypergeometric FDR < 0.05 and enrichment > 2, compared with background; Tables S3 and S4, Supplemental Experimental Procedures). These include diabetes-relevant terms such as maturity-onset diabetes of the young (MODY), a form of monogenic diabetes caused mainly by mutations in islet transcription factors (Murphy et al., 2008). In addition, terms associated with glucose sensing and insulin metabolism and secretion were identified, as well as those relating to endocrine pancreas fate decisions and beta-cell development. For instance, *FOXA2* (Lee et al., 2002), *NKX2-2* (Sussel et al., 1998), and *PDX1* (Stoffers et al., 1997) were highlighted, each of these genes serving important roles in islet development. WNT and NOTCH signaling terms were also enriched; these pathways are important in the induction of posterior endoderm and pancreatic progenitors, as well as pancreatic endocrine versus exocrine fate choices (Cras-Meneur et al., 2009;

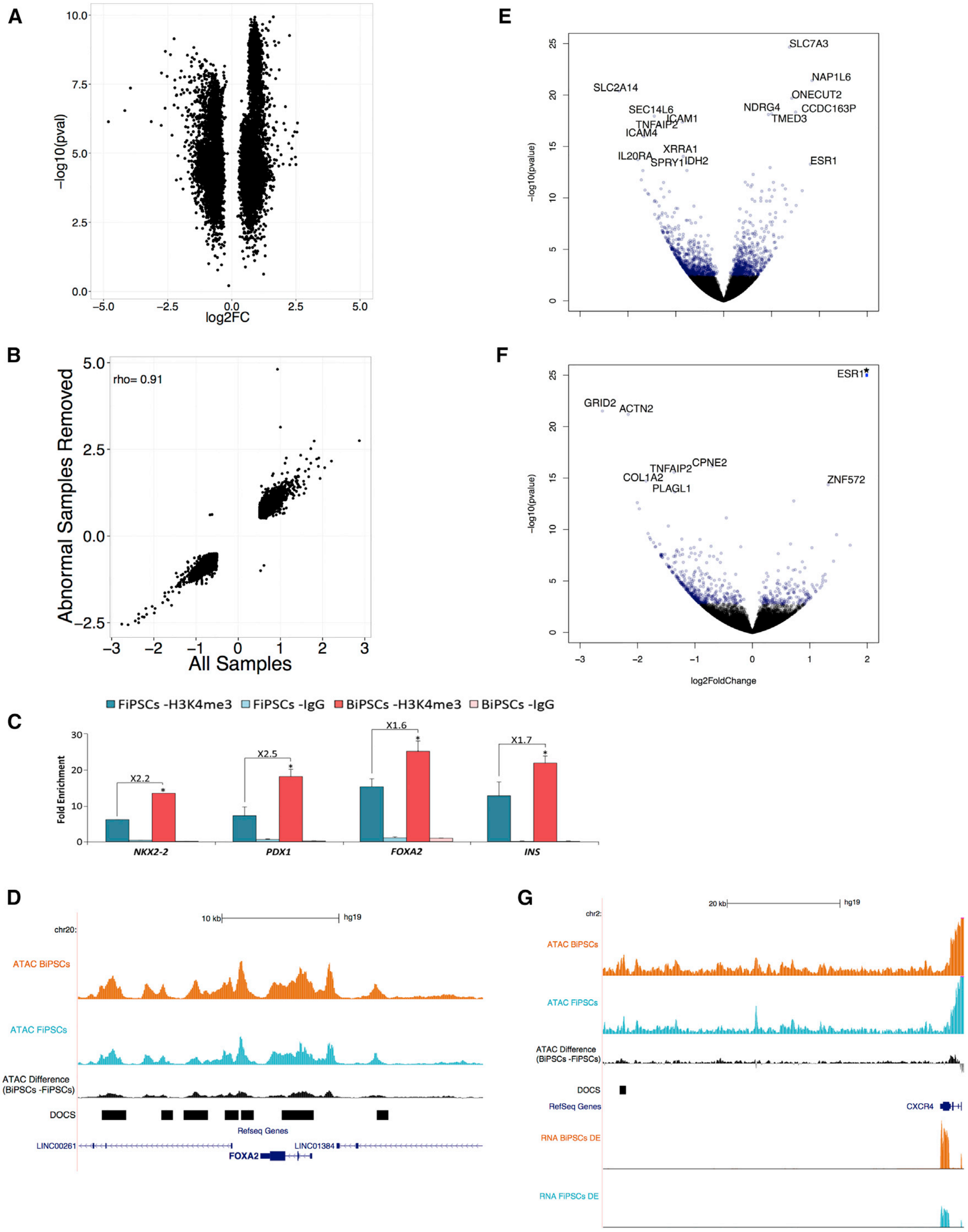

Figure 3. Validation of DOCS

(A) DOCS Volcano plot with log2FC (x axis) and −log10 p value (y axis) of karyotypically normal samples only.

(B) Log2FC correlation of overlapping DOCS identified from all samples (x axis) or only karyotypically normal samples (y axis).

(C) ChIP-qPCR analysis of H3K4me3 in BiPSCs (n = 5) and FiPSCs (n = 4). Values are means ± SEM and normalized to *CRYAA* and *TEX15*. $^*p < 0.05$.

(D) *FOXA2* region with normalized read depth of BiPSCs (orange) and FiPSCs (blue) samples. In black, ATAC read depth difference between iPSC types. Bottom tracks: DOCS (black bar) and genes (blue).

(legend continued on next page)

Nostro et al., 2011). Multiple terms relating to neuronal development were also enriched; this is consistent with gene expression profiles observed between developing pancreatic islets and neurons (van Arensbergen et al., 2010).

To evaluate signatures of epigenetic memory, which could explain the enhanced endodermal differentiation capacity of BiPSCs, we tested whether Bi-DOCS-associated genes relevant for endodermal development were also open in human islets. We found that 99% of Bi-DOCS-associated genes, which are specifically expressed during endocrine pancreas development (van de Bunt et al., 2016), were also associated with open chromatin in human islets. In addition, when comparing the total number of Bi-DOCS and adult islet open chromatin-associated genes, we found that Bi-DOCS-associated genes were significantly enriched in stage-specific endocrine pancreas developmental genes versus those associated with adult islet open chromatin (Fisher test odds ratio [OR] = 1.3, $p = 1.9 \times 10^{-13}$).

The overlap of Bi-DOCS-associated genes with those open in human islets suggests BiPSCs could retain an epigenetic signature of their cell type of origin. In addition, the enrichment of Bi-DOCS for endocrine pancreas developmental stage-specific genes could explain the enhanced propensity of BiPSCs to differentiate down this same developmental lineage.

In Silico and Cellular Validation of DOCS

Using random permutations of sample labels to account for biases caused by donor-dependent factors, we found that the number of DOCS identified in permuted samples was lower than that observed in the original analysis (mean random sites = 2.0k versus 8.3k in the original analysis). This confirms that donor-specific factors are not the major driver in the differential open chromatin analysis in our study. Furthermore, we found that removing samples with an abnormal karyotype (lines BiPSC-D 1–2) yielded results similar to the original analysis (at 4.8k/8.3k overlapping DOCS) including all samples (Figures 3A and 3B, Supplemental Experimental Procedures).

ATAC-seq data and prediction of Bi-DOCS were validated *in vitro* using H3K4me3 chromatin immunoprecipitation (ChIP)-qPCR and primers flanking a subset of DOCS near the promoters of *PDX1*, *NKX2-2*, *FOXA2*, and *INS*; these genes highlighted by the gene enrichment analysis. All five BiPSC lines showed higher chromatin enrichment in the promoter regions of *PDX1*, *NKX2-2*, *FOXA2,* and *INS* versus FiPSCs (Figure 3C). Figure 3D visualizes seven DOCS between FiPSCs and BiPSCs around the *FOXA2* promoter.

Bi-DOCS Affect Gene Expression at Key Stages of Islet Development

To confirm that Bi-DOCS contain regulatory annotations that have an impact on gene expression during development, we performed directed differentiation of a subset of FiPSCs and BiPSCs cell lines toward islet-like cells and collected RNA at two key developmental stages: definitive endoderm (DE) and pancreatic progenitors (PP) (Table S1). RNA-seq and differential expression analysis identified 1,247 protein-coding genes differentially expressed (FDR < 0.05) between BiPSCs- and FiPSCs-derived cells at the DE stage (567 genes upregulated in BiPSC-derived and 680 in FiPSC-derived cells, Figure 3E), and 607 genes at the PP stage (181 genes upregulated in BiPSC-derived cells, 426 in FiPSC-derived cells, Figure 3F). Genes upregulated in BiPSCs-derived cells at both stages were significantly enriched in those mapping to Bi-DOCS (hypergeometric $FDR_{DE} = 3.4 \times 10^{-7}$, $FDR_{pp} = 9.1 \times 10^{-4}$), while genes upregulated in FiPSC-derived cells were significantly enriched in Fi-DOCS genes (hypergeometric $FDR_{DE} = 2.5 \times 10^{-14}$, $FDR_{pp} = 5.0 \times 10^{-8}$, Table S6). We detected a proximal Bi-DOCS site for 126 of the 567 genes upregulated at BiPSC-derived DE, and a Fi-DOCS site for 145 of the 680 genes upregulated at FiPSC-derived DE. Bi-DOCS-associated genes include those of known endoderm developmental biology, such as *CXCR4* (Katsumoto and Kume, 2011). *CXCR4* was significantly upregulated in BiPSCs at the DE stage (log2FC = 1.01, $p_{adj} = 7.4 \times 10^{-6}$, Figure 3G) and has a Bi-DOC site that overlaps a DE weak enhancer; a type of chromatin state involved in endodermal development (Wang et al., 2015).

Finally, genes upregulated in BiPSCs at the DE stage were significantly enriched for FOXA2 target genes expressed at this stage (hypergeometric $FDR = 1.4 \times 10^{-53}$, Table S6) which is in line with the FOXA2 TFBS enrichment in Bi-DOCS.

DISCUSSION

Here, we systematically cataloged sites of open chromatin across the genome of BiPSCs to explain the preferential endodermal lineage commitment of BiPSCs versus FiPSCs. We were able to identify Bi-DOCS and showed that these

(E and F) Volcano plot with log2FC (x axis) and −log10 p value (y axis) of differentially expressed genes between FiPSC- and BiPSC-derived DE (E) and PP (F) cells. **ESR1* at PP stage out-of-scale in (F) (true log2FC = 5.74, −log10 p value = 100.9).
(G) *CXCR4* region comparing normalized ATAC-seq read depth of BiPSCs (orange) and FiPSCs (blue, top). In black (middle), read depth difference between BiPSCs and FiPSCs. DOCS (black bar) are shown in the middle. Bottom tracks: *CXCR4* RNA-seq data of BiPSC- (orange) and FiPSC (blue)-derived DE cells.

Bi-DOCS were enriched for genomic regulatory annotations such as weak enhancers, bivalent enhancers, bivalent promoters, and FOXA2 binding sites shown to be active during endoderm lineage commitment (Wang et al., 2015; Xie et al., 2013).

We also confirmed that Bi-DOCS could be linked to gene expression changes at two stages of *in vitro* differentiation into islet-like cells. We also observed a significant enrichment of the FOXA2 targets reported at the DE stage within the genes upregulated in BiPSCs at this stage. FOXA2 is one of the master regulators of endodermal development (Raum et al., 2006), with FOXA2 binding sites being enriched at endodermal poised weak enhancers, thereby priming cells to receive extracellular cues for differentiation into endodermal lineage organs (Wang et al., 2015). Bi-DOCS were also enriched for GO terms relating to early pancreatic endoderm signaling pathways (Nostro et al., 2015; Pagliuca et al., 2014; Rezania et al., 2014), as well as MODY genes. This suggests that Bi-DOCS represent signatures of epigenetic memory and drive the differences in gene expression, which may account for the enhanced propensity of BiPSCs to differentiate toward endodermal lineage. Bi-DOCS may thus provide clues to genes and pathways involved in islet-lineage development.

The extent of epigenetic "memory" in reprogrammed iPSCs remains a subject of debate. Alongside data suggesting donor genotype may drive differentiation potential (Burrows et al., 2016; Kyttala et al., 2016; Rouhani et al., 2014), technical artifacts such as culture conditions, cell-cycle phase, and reprogramming technology may also give rise to transcriptional and epigenetic differences between iPSC lines. Additional work will be required to determine whether the differences reported here are solely dependent on the donor cell type. Specifically, the two iPSC types in our study differ substantially in passage number, and prolonged passaging has been suggested to eradicate iPSC epigenetic memory (Polo et al., 2010).

Future work should focus on increasing sample number and integrating our data with additional genomic annotations to further elucidate the mechanisms driving developmental competence and differentiation capacity. In conclusion, our findings provide a valuable resource for improving endocrine pancreas lineage differentiation protocols and may lead to the development of enhanced islet cell models, and ultimately improved therapeutic approaches for diabetes.

EXPERIMENTAL PROCEDURES

iPSC Generation and Human Islet Sample Collection

As described in Supplemental Experimental Procedures, relevant Ethics and Institutional Review Boards (IRBs) approved the use of human islets and human iPSCs in this study. BiPSC and FiPSC lines were generated by reprogramming beta cells and skin fibroblasts as described previously (Bar-Nur et al., 2011; van de Bunt et al., 2016). Five human islets were freshly isolated from cadaveric donors as previously described (van de Bunt et al., 2016). All samples were processed for ATAC-seq as described in the Supplemental Experimental Procedures.

Computational and Statistical Analysis

ATAC-seq and RNA-seq reads were processed and aligned to the genome (build hg19). We then predicted open chromatin regions, identified DOCS, and differentially expressed genes as described in the Supplemental Experimental Procedures. Statistical analysis was performed in R version 3.0.2 unless stated otherwise. Data have been deposited in public repositories (Supplemental Experimental Procedures).

ACCESSION NUMBERS

Data have been deposited at the EBI hosted European Genome-phenome Archive (EGA, http://www.ebi.ac.uk/ega/) and European Nucleotide Archive (ENA, http://www.ebi.ac.uk/ena). EGA: EGAS00001002591 (islet ATAC-seq and iPSC RNA-seq). ENA: PRJEB21856 (iPSC ATAC-seq).

SUPPLEMENTAL INFORMATION

Supplemental Information includes Supplemental Experimental Procedures, two figures, and six tables and can be found with this article online at https://doi.org/10.1016/j.stemcr.2017.09.020.

AUTHOR CONTRIBUTIONS

M.T., L.S., A.L.G., M.I.M., N.L.B., and S.E. designed the study. A.B. and A.J.B. obtained the human islet samples and performed quality control. L.S. and N.L.B. performed iPSC experiments. M.T. performed bioinformatics analysis of ATAC-seq data. A.W.-A. analyzed the RNA-seq data. M.T., L.S., and N.L.B. wrote the manuscript. A.W.A., M.I.M., A.L.G., and S.E. gave conceptual advice and edited the manuscript.

ACKNOWLEDGMENTS

We thank the High-Throughput Genomics Group at the Wellcome Trust Centre for Human Genetics (funded by Wellcome Trust grant 090532) for the generation of the sequencing data. We thank Tamar Golan-Lev and Nissim Benvenisty for karyotyping the BiPSC lines. The research here received support from the Innovative Medicines Initiative Joint Undertaking under grant agreement no. 115439, resources of which are composed of financial contribution from the European Union's Seventh Framework Program (FP7/2007–2013) and EFPIA companies in kind contribution. This publication reflects only the authors' views and neither the IMI JU nor EFPIA nor the European Commission is liable for any use that may be made of the information contained therein. This work was also supported by the Wellcome Trust (099673; 095101; 200837; 098381; 1060130; 099673/Z/12/Z), the Medical Research Council (MR/L020149/1), and the National Institute for Health Research (NIHR) Oxford Biomedical Research Centre

Program. A.L.G. is a Wellcome Trust Senior Fellow in Basic Biomedical Research; M.I.M. is a Wellcome Trust Senior Investigator. N.L.B. was a Naomi Berrie Fellow in Diabetes Research and is now an employee of Novo Nordisk (although all experimental work was carried out under employment at University of Oxford). M.T. was supported by a Wellcome Trust Doctoral Studentship. This study was performed as part of the requirements for the PhD thesis of L.S.

Received: February 6, 2017
Revised: September 22, 2017
Accepted: September 25, 2017
Published: October 26, 2017

REFERENCES

Balboa, D., and Otonkoski, T. (2015). Human pluripotent stem cell based islet models for diabetes research. Best Pract. Res. Clin. Endocrinol. Metab. *29*, 899–909.

Bar-Nur, O., Russ, H.A., Efrat, S., and Benvenisty, N. (2011). Epigenetic memory and preferential lineage-specific differentiation in induced pluripotent stem cells derived from human pancreatic islet beta cells. Cell Stem Cell *9*, 17–23.

Burrows, C.K., Banovich, N.E., Pavlovic, B.J., Patterson, K., Gallego Romero, I., Pritchard, J.K., and Gilad, Y. (2016). Genetic variation, not cell type of origin, underlies the majority of identifiable regulatory differences in iPSCs. PLoS Genet. *12*, e1005793.

Cras-Meneur, C., Li, L., Kopan, R., and Permutt, M.A. (2009). Presenilins, Notch dose control the fate of pancreatic endocrine progenitors during a narrow developmental window. Genes Dev. *23*, 2088–2101.

Dimas, A.S., Lagou, V., Barker, A., Knowles, J.W., Magi, R., Hivert, M.F., Benazzo, A., Rybin, D., Jackson, A.U., Stringham, H.M., et al. (2014). Impact of type 2 diabetes susceptibility variants on quantitative glycemic traits reveals mechanistic heterogeneity. Diabetes *63*, 2158–2171.

Encode Project Consortium (2012). An integrated encyclopedia of DNA elements in the human genome. Nature *489*, 57–74.

Katsumoto, K., and Kume, S. (2011). Endoderm and mesoderm reciprocal signaling mediated by CXCL12 and CXCR4 regulates the migration of angioblasts and establishes the pancreatic fate. Development *138*, 1947–1955.

Kim, K., Doi, A., Wen, B., Ng, K., Zhao, R., Cahan, P., Kim, J., Aryee, M.J., Ji, H., Ehrlich, L.I., et al. (2010). Epigenetic memory in induced pluripotent stem cells. Nature *467*, 285–290.

Kundaje, A., Meuleman, W., Ernst, J., Bilenky, M., Yen, A., Heravi-Moussavi, A., Kheradpour, P., Zhang, Z., Wang, J., Ziller, M.J., et al. (2015). Integrative analysis of 111 reference human epigenomes. Nature *518*, 317–330.

Kyttala, A., Moraghebi, R., Valensisi, C., Kettunen, J., Andrus, C., Pasumarthy, K.K., Nakanishi, M., Nishimura, K., Ohtaka, M., Weltner, J., et al. (2016). Genetic variability overrides the impact of parental cell type and determines iPSC differentiation potential. Stem Cell Reports *6*, 200–212.

Lee, C.S., Sund, N.J., Vatamaniuk, M.Z., Matschinsky, F.M., Stoffers, D.A., and Kaestner, K.H. (2002). Foxa2 controls Pdx1 gene expression in pancreatic beta-cells in vivo. Diabetes *51*, 2546–2551.

Murphy, R., Ellard, S., and Hattersley, A.T. (2008). Clinical implications of a molecular genetic classification of monogenic beta-cell diabetes. Nat. Clin. Pract. Endocrinol. Metab. *4*, 200–213.

Nostro, M.C., Sarangi, F., Ogawa, S., Holtzinger, A., Corneo, B., Li, X., Micallef, S.J., Park, I.H., Basford, C., Wheeler, M.B., et al. (2011). Stage-specific signaling through TGFbeta family members and WNT regulates patterning and pancreatic specification of human pluripotent stem cells. Development *138*, 861–871.

Nostro, M.C., Sarangi, F., Yang, C., Holland, A., Elefanty, A.G., Stanley, E.G., Greiner, D.L., and Keller, G. (2015). Efficient generation of NKX6-1+ pancreatic progenitors from multiple human pluripotent stem cell lines. Stem Cell Reports *4*, 591–604.

Pagliuca, F.W., Millman, J.R., Gurtler, M., Segel, M., Van Dervort, A., Ryu, J.H., Peterson, Q.P., Greiner, D., and Melton, D.A. (2014). Generation of functional human pancreatic beta cells in vitro. Cell *159*, 428–439.

Pasquali, L., Gaulton, K.J., Rodriguez-Segui, S.A., Mularoni, L., Miguel-Escalada, I., Akerman, I., Tena, J.J., Moran, I., Gomez-Marin, C., van de Bunt, M., et al. (2014). Pancreatic islet enhancer clusters enriched in type 2 diabetes risk-associated variants. Nat. Genet. *46*, 136–143.

Polo, J.M., Liu, S., Figueroa, M.E., Kulalert, W., Eminli, S., Tan, K.Y., Apostolou, E., Stadtfeld, M., Li, Y., Shioda, T., et al. (2010). Cell type of origin influences the molecular and functional properties of mouse induced pluripotent stem cells. Nat. Biotechnol. *28*, 848–855.

Raum, J.C., Gerrish, K., Artner, I., Henderson, E., Guo, M., Sussel, L., Schisler, J.C., Newgard, C.B., and Stein, R. (2006). FoxA2, Nkx2.2, and PDX-1 regulate islet beta-cell-specific mafA expression through conserved sequences located between base pairs -8118 and -7750 upstream from the transcription start site. Mol. Cell. Biol. *26*, 5735–5743.

Rezania, A., Bruin, J.E., Arora, P., Rubin, A., Batushansky, I., Asadi, A., O'Dwyer, S., Quiskamp, N., Mojibian, M., Albrecht, T., et al. (2014). Reversal of diabetes with insulin-producing cells derived in vitro from human pluripotent stem cells. Nat. Biotechnol. *32*, 1121–1133.

Rouhani, F., Kumasaka, N., de Brito, M.C., Bradley, A., Vallier, L., and Gaffney, D. (2014). Genetic background drives transcriptional variation in human induced pluripotent stem cells. PLoS Genet. *10*, e1004432.

Stoffers, D.A., Zinkin, N.T., Stanojevic, V., Clarke, W.L., and Habener, J.F. (1997). Pancreatic agenesis attributable to a single nucleotide deletion in the human IPF1 gene coding sequence. Nat. Genet. *15*, 106–110.

Sussel, L., Kalamaras, J., Hartigan-O'Connor, D.J., Meneses, J.J., Pedersen, R.A., Rubenstein, J.L., and German, M.S. (1998). Mice lacking the homeodomain transcription factor Nkx2.2 have diabetes due to arrested differentiation of pancreatic beta cells. Development *125*, 2213–2221.

van Arensbergen, J., Garcia-Hurtado, J., Moran, I., Maestro, M.A., Xu, X., Van de Casteele, M., Skoudy, A.L., Palassini, M., Heimberg, H., and Ferrer, J. (2010). Derepression of Polycomb targets during

pancreatic organogenesis allows insulin-producing beta-cells to adopt a neural gene activity program. Genome Res. *20*, 722–732.

van de Bunt, M., Lako, M., Barrett, A., Gloyn, A.L., Hansson, M., McCarthy, M.I., Beer, N.L., and Honore, C. (2016). Insights into islet development and biology through characterization of a human iPSC-derived endocrine pancreas model. Islets *8*, 83–95.

ViaCyte. (2014). A Safety, Tolerability, and Efficacy Study of VC-01™ Combination Product in Subjects with Type I Diabetes Mellitus. https://clinicaltrials.gov/ct2/show/NCT02239354.

Wang, A., Yue, F., Li, Y., Xie, R., Harper, T., Patel, N.A., Muth, K., Palmer, J., Qiu, Y., Wang, J., et al. (2015). Epigenetic priming of enhancers predicts developmental competence of hESC-derived endodermal lineage intermediates. Cell Stem Cell *16*, 386–399.

Xie, R., Everett, L.J., Lim, H.W., Patel, N.A., Schug, J., Kroon, E., Kelly, O.G., Wang, A., D'Amour, K.A., Robins, A.J., et al. (2013). Dynamic chromatin remodeling mediated by polycomb proteins orchestrates pancreatic differentiation of human embryonic stem cells. Cell Stem Cell *12*, 224–237.

Stem Cell Reports, Volume *9*

Supplemental Information

Genes Associated with Pancreas Development and Function Maintain Open Chromatin in iPSCs Generated from Human Pancreatic Beta Cells

Matthias Thurner, Liraz Shenhav, Agata Wesolowska-Andersen, Amanda J. Bennett, Amy Barrett, Anna L. Gloyn, Mark I. McCarthy, Nicola L. Beer, and Shimon Efrat

Tissue	Donor ID	Sample ID	Passage #	Karyotype	ATAC-seq	RNA-seq
Fibroblast derived	Individual A	FiPSC_A.1	24-25	46XX	✓	DE+PP stage
		FiPSC_A.2	24-26	46XX	✓	DE+PP stage
		FiPSC_A.3	21	46XX	✓	NA
	Individual B	FiPSC_B.1	20-26	46XY	✓	DE+PP stage
		FiPSC_B.2	18-24	46XY	✓	DE+PP stage
Beta-cell derived	Individual C	BiPSC_C.1	11-12	46XX	✓	DE+PP stage
		BiPSC_C.2	10-12	46XX	✓	DE+PP stage
	Individual D	BiPSC_D.1	9-10	47XY+20	✓	DE+PP stage
		BiPSC_D.2	10	47XY+20	✓	NA
	Individual E	BiPSC_E.1	9-11	46XX	✓	DE+PP stage

Table S1, related to Figure 1. Sample characteristics. RNA-seq data for each stage was collected in duplicates. Abbreviations: Definitive Endoderm (DE); Pancreatic Progenitor (PP)

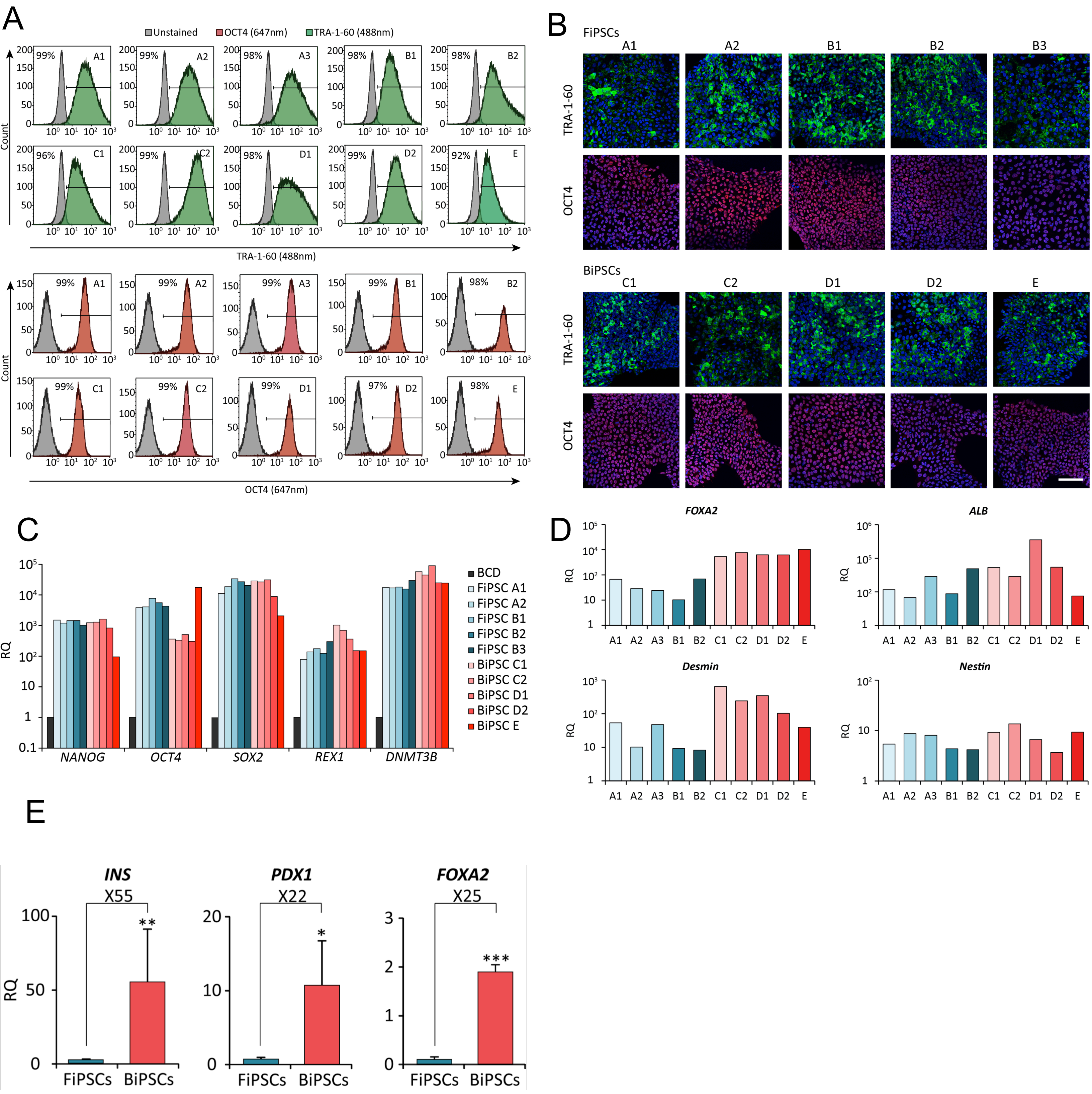

Figure S1, related to Figure 1. iPSC lines characterisation. **A:** FACS analysis of TRA-1-60 and OCT4 in FiPSCs (A1-B2) and BiPSCs (C1-E) clones. **B** Immunofluorescence analysis of TRA-1-60 and OCT4 in iPSC clones. Scale bar=100µm. **C:** qPCR analysis of pluripotency gene expression in iPSC lines in comparison to BCDs (RQ=1). Results were normalized to GAPDH. **D**: qPCR analysis of representative markers from the three embryonic germ layers in EBs differentiated from BiPSCs. FOXA2 (endoderm), ALB (endoderm), Desmin (mesoderm) and Nestin (ectoderm). Values are compared to undifferentiated hESC-H1 (RQ=1) and are normalized to GAPDH. **E:** qPCR analysis of *INS*, *PDX1* and *FOXA2* transcripts in 21-day EBs derived from BiPSCs (n=5) and FiPSCs (n=5), in comparison to EBs derived from hESC- H1 (RQ=1). Values are mean±SE and are normalized to GAPDH. *P- value<0.05; **P-value-<0.001, ***P- value-<0.0001.

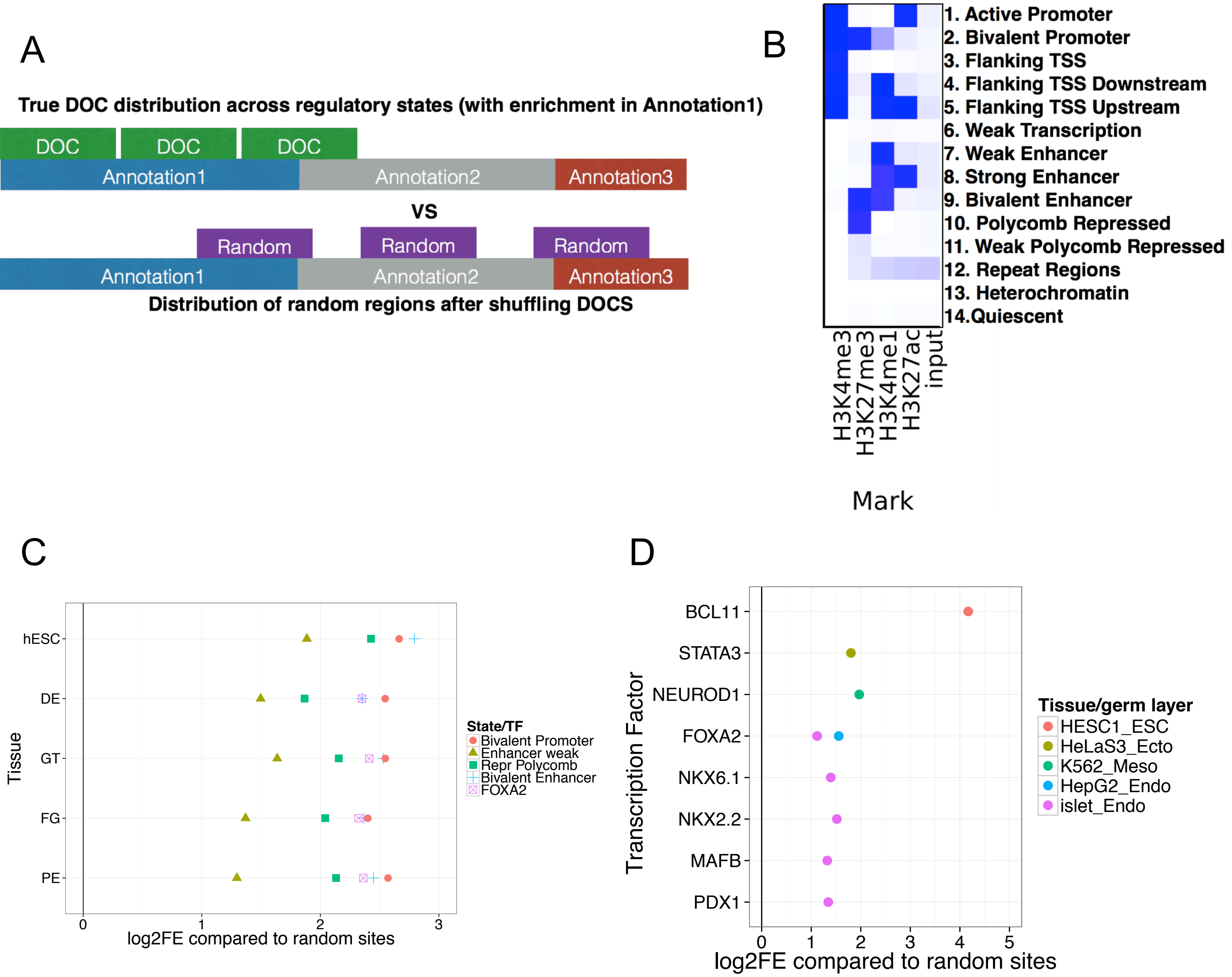

Figure S2, related to Figure 2. DOCS enrichment across various regulatory annotations compared to random sites. A) Schematic diagram of evaluating enrichment of DOCS in regulatory annotations compared to randomly shuffled regions. B) 14 State ChromHMM model predicted across stages of endodermal development using the indicated histone marks derived from Wang et al 2015 and Xie et al 2013. The colour is based on the chromHMM emission parameters and a darker colour indicates a higher frequency of a mark at a given state. C) Log2FE of DOCS (x-axis) in chromatin states and FOXA2 TFBS (indicated by shape and colour) across stages of endodermal development (y-axis). hESC (Human Stem cell, not available for FOXA2), Definitive Endoderm (DE), Gut Tube (GT), Fore Gut (FG) and Pancreatic Endoderm (PE). D) Log2FE of DOCS (x-axis) in HESC-H1 (stem cell), HeLaS3 (ectoderm), K562 (mesoderm), HepG2 (endoderm) and islet (endoderm) TFBS (y-axis) compared to random sites. Colours indicate different cell types.

Lineage tracing primers		
	Fw	Rev
**loxp's flanked**	ATCCACGCTGTTTTGACCTC	AAGTCGTGCTGCTTCATGTG

Primer sets for RNA qPCR		
	Fw	Rev
**OCT4**	AGTTTGTGCCAGGGTTTTTG	ACTTCACCTTCCCTCCAACC
**SOX2**	GGGAAATGGGAGGGGTGCAAAAGAGG	TTGCGTGAGTGTGGATGGGATTGGTG
**NANOG**	TTTGGAAGCTGCTGGGGAAG	GATGGGAGGAGGGGAGAGGA
**DNMT3B**	ATAAGTCGAAGGTGCGTCGT	GGCAACATCTGAAGCCATTT
**REX1**	TCGCTGAGCTGAAACAAATG	CCCTTCTTGAAGGTTTACAC
**GAPDH**	GTGGACCTGACCTGCCGTCT	GGAGGAGTGGGTGTCGCTGT
**PDX1**	Taqman probe Hs_00426216_m1	Taqman probe Hs_00426216_m1
**INS**	Taqman probe Hs 00355773 m1	Taqman probe Hs 00355773 m1
**FOXA2**	Taqman probe Hs_00232764_m1	Taqman probe Hs_00232764_m1
**AFP**	Taqman probe Hs_00173490_m1	Taqman probe Hs_00173490_m1
**ALB**	Taqman probe Hs_00609403_m1	Taqman probe Hs_00609403_m1
**DES**	Taqman probe Hs_00157258_m1	Taqman probe Hs_00157258_m1
**GAPDH**	Taqman probe Hs_99999905_m1	Taqman probe Hs_99999905_m1

Primer sets for ChIP-qPCR		
	Fw	Rev
**FOXA2**	TTCTTCGCTCTCAGTGCTCA	TCCGGGTCTGAACTGTAACA
**NKX2-2**	GACGACATTAACGCTGGGAC	GTCTCCTTGGAGTGGCAGAT
**INS**	GCCAGAGGAGAGAGGATCAG	TATGCAAGTCCAACGCACTG
**PDX1**	GTGGGTTCCCTCTGAGATCA	GTCCTTGTAAAGCTGCGTGG
**APRT**	GCCTTGACTCGCACTTTTGT	TAGGCGCCATCGATTTTAAG
**TEX15**	TACATTGCCCTGCCATGGTA	GCCTCCTCCTCAGAAGTTGT
**CRYAA**	TCCACCATCAGCCCCTACTA	GCCACGTCTTACCTCAGAGA

Table S5, related to Experimental Procedures. Primer sets used in the analysis. Primer sets, which were used for lineage tracing (top) and qPCR for both RNA (middle) and ChIP (bottom), are shown.

Stage	Gene direction of effect	BiDOCS	FiDOCS	DE FOXA2 targets
DE	Up in BiPSC-derived cells	**3.4E-07**	0.63	**1.4E-53**
DE	Up in FiPSC-derived cells	0.09	**2.5E-14**	1.0
PP	Up in BiPSC-derived cells	**9.1E-04**	0.96	**1.6E-03**
PP	Up in FiPSC-derived cells	**0.02**	**5.0E-08**	1.0

Table S6, related to Figure 3. Enrichment of DE and PE differentially expressed genes (hypergeometric FDR values) in Bi-DOCS and Fi-DOCS associated genes as well as active DE FOXA2 target genes. Significant enrichment (FDR<0.05) is highlighted in bold.

2. Supplemental Experimental Procedures

2.1 iPSC generation and characterization

Human pancreatic islets were received 2-4 days following isolation, dissociated into single cells, labelled for beta-cell lineage tracing and cultured as described (Russ et al., 2008). Labelled Beta-Cell-Derived (BCD) cells were sorted using a FACS Aria cell sorter (Becton Dickinson, San Jose, CA). BiPSCs were generated as previously described (Bar-Nur et al., 2011) with the following modification: sorted BCD cells were expanded to passage 8-16 and transduced with a polycistronic lentiviral vector containing the four reprogramming transcription factors OCT4, SOX2, KLF4 and c-MYC (a gift of Gustavo Mostoslavsky). ES-like colonies emerged 3-7 weeks following transduction. Five of these colonies, originating from three different human donors, were isolated and expanded for further analysis. The beta-cell origin of iPSC clones (BiPSCs) was validated by DNA PCR analysis of the recombined reporter cassette as previously described (Bar-Nur et al., 2011). BiPSC pluripotency was confirmed via FACS staining for TRA-1-60 (Biolegend, 330614) and OCT4 (BD Biosciences, 560329) using a GalliosTM Flow Cytometer (Beckman Coulter Life Sciences; 20,000–50,000 events recorded per sample).

iPSCs reprogrammed from human fibroblasts (FiPSCs) were obtained through the IMI/EU sponsored StemBANCC consortium via the Human Biomaterials Resource Centre, University of Birmingham and were generated as described previously (van de Bunt et al., 2016).

H1-hESC line was a gift of Joseph Itskovitz-Eldor. The StemBANCC consortium did not fund work associated with the H1-hESC line and the cell line was only used for comparison of the qPCR RNA expression analysis described below. FiPSC pluripotency was confirmed via FACS as before. Karyotypic QC involved: for BiPSCs G-banding as described (Bar-Nur et al., 2011), or for FiPSCs Illumina Human CytoSNP-12v2.1 beadchips (Illumina, WG-320).
iPSCs and H1-hESC cells were cultured on Matrigel™ hESC-qualified Matrix (BD Biosciences 354277) with mTeSR™1 medium (Stem Cell Technologies 05850). Embryoid bodies were generated as described previously (Bar-Nur et al., 2011). Total RNA was extracted using ZR RNA MiniPrep Kit and treated with DNase according to the manufacturer's instruction (Zymo Research, R1065). cDNA was produced by reverse transcription using High-Capacity cDNA RT Kit (Applied Biosystems, 4368813) or qScript cDNA synthesis kit (Quanta, 95047). qPCR was carried out in a 7300 real-time PCR instrument (Applied Biosystems), using FAST SYBR Green or TaqMan Universal PCR Master Mix (Applied Biosystems) with primers listed in Table S5.

Ethics statement

This study was conducted according to the principles expressed in the Declaration of Helsinki. The Institutional Review Boards of the following medical centres, which provided human islets, each provided approval for the collection of samples and subsequent analysis: University of Geneva School of Medicine; University of Minnesota; Invention Institute California. All donors provided written informed consent for the collection of all samples and subsequent analysis.
Skin fibroblast samples for reprogramming were collected with full informed consent. Ethical approval for the StemBANCC study (UK) was received from the National Research Ethics Service South Central Hampshire A research ethics committee (REC 13/SC/0179).

2.2 Human islets (from Oxford) used for ATAC-seq

Human islets were freshly isolated from cadaveric donors at the Oxford Centre for Islet Transplantation as described previously (Cross et al., 2012; van de Bunt et al., 2015) and processed for ATAC-seq (Buenrostro et al., 2013) after 1-3 days of storage in either UW (Belzer) or CMRL (without glutamine from Life Technologies) media.

Ethics Statement for the Oxford human islets

Human islet studies were approved by the Human Research Ethics Board the University of Oxford's Oxford Tropical Research Ethics Committee (OxTREC Reference: 2–15)

2.3 ATAC-seq

ATAC-seq was performed as described previously (Buenrostro et al., 2013) using ~50,000 cells per sample. Briefly, samples were lysed and transposed using T5 transposases (Illumina, FC-121-1030) and PCR (New England Labs Cat, M0541) amplified for 11 cycles to generate sequencing libraries. Libraries were purified using the Quiagen Minelute kit (28004) and Beckman Coulter Agencourt

AMPure beads (A63880) to remove primer dimers. The resulting libraries were multiplexed using primers Ad_1-6 (described in Buenrostro et al., 2013) and sequenced across 1-2 lanes of Hiseq2500 (Illumina).

2.4 Mapping and filtering of ATAC-seq reads

Raw FASTQ reads were processed with an in-house pipeline first described in (Hay et al., 2016) and on the following website http://userweb.molbiol.ox.ac.uk/public/telenius/PipeSite.html. Specifically, library and sequencing quality was checked with FASTQC (http://www.bioinformatics.babraham.ac.uk/projects/fastqc/) and reads were mapped to the human genome hg19 via bowtie version 1.1.0 (Langmead et al., 2009) with default settings but -m 2, and maxins 2000. For reads that could not be aligned the first time, adapters were removed with Trim Galore (http://www.bioinformatics.babraham.ac.uk/projects/trim_galore/) at the 3 prime end (settings -length 10, -qualFilter 20) to enhance the chance of mapping. The resulting trimmed reads were then mapped again with bowtie. Any remaining unmapped and trimmed reads were processed with FLASH version 1.2.8 settings -m 9 -x 0.125 (Magoc and Salzberg, 2011). Since overlapping paired-end reads can often not be mapped by alignment software, FLASH combines the read pair and reconstructs a read pair without overlap. These are then realigned a third time using bowtie. PCR duplicates are then removed from the mapped bam files using samtools rmdup function (Li et al., 2009). All reads overlapping any of the "unmappable" UCSC Duke blacklisted hg19 regions (https://sites.google.com/site/anshulkundaje/projects/blacklists) were also removed from the final bam file.

2.5 Peak calling and normalisation of reads

Peaks were called from filtered bam files using MACS2 (settings FDR <0.01, –nomodel, -g hs, (Zhang et al., 2008)), so as to identify regions of open chromatin. Peaks were merged for each cell type separately (human pancreatic islets, FiPSCs and BiPSC) using bedtools (Quinlan and Hall, 2010) mergeBed function. Read depth across peaks was normalised by applying the bamnormalise and bamsummary function of the software package deeptools (Ramirez et al., 2016) using standard settings.

2.6 Identification of DOCS and genomic annotations

Identification of DOCS was performed using 1000bp sliding window approach with 100bp step-size and a negative binomial test as implemented in the perl software diffReps (Shen et al., 2013). After analysing the whole genome, overlapping windows with a significant difference in normalised read depth (predefined as P<0.0001) were merged together, and then retested. Significant DOCS with an absolute minimum log2FC of 0.5 were identified after performing multiple testing correction using an FDR (FDR<0.05) approach, this based on the number of merged and retested regions. Standard settings were used apart from a defined fragment size of 50bp.

2.7 Using Epigenome Roadmap states and other epigenomic annotations to interrogate the role of DOCS

We obtained Expanded 18-state chromatin states from the Epigenome Roadmap data repository (http://egg2.wustl.edu/roadmap/web_portal/chr_state_learning.html#exp_18state). We permuted DOCS by shuffling them 1000 times using bedtools shuffleBed function, this distributing DOCS randomly across the genome. For the 98 Epigenome chromHMM states, DOCS enrichment was calculated in the following way: Each individual DOC site has an associated log2FC value indicating the difference in read depth between BiPSCs and FiPSCs. We estimated enrichment by determining the sum of log2FC for each type of chromatin state based on all true DOCS. This true value of each chromatin state was then compared to the mean of the sum of log2FC for each chromatin state derived from 1000 permutations of shuffled DOCS (true DOCS were shuffled using the shuffleBed function implemented in bedtools). For each state and each of the 98 cell types, P-values were calculated based on how often the shuffled sum of log2FC was higher compared to the true sum of log2FC (minimum P-value 0.001). The average P-value for each of the 6 germ layer/pluripotent cell types (Ectoderm, Endoderm, Mesoderm, ESC, iPSC and Others) was calculated across all cells of a given type and chromatin state. These averaged P-values were FDR corrected and used for the analysis.

We also obtained information for ChIP-seq marks from a previously-published model of endocrine pancreas development (Wang et al., 2015; Xie et al., 2013), including: H3K4me3, H3K27ac, H3K4me1, H3K27me3, Input control and FOXA2. Specifically, this model included cells that represented the following islet developmental stages: human stem cells (HSC/iPSC), Definitive

Endoderm (DE), Primitive Gut Tube (PGT), Posterior ForeGut (PFG), and Pancreatic Endoderm (PE). After aligning the FASTQ reads to the genome and filtering duplicated reads using picard tools (v1.119, http://broadinstitute.github.io/picard/), we used this histone mark information to identify chromatin regulatory states via chromHMM as described previously (Ernst and Kellis, 2012). Specifically, we identified 14 chromatin regulatory states, including bivalent enhancers (H3K27me3 and H3K4me1) and promoters (H3K4me3 and H3K27me3), weak enhancers (H3Kme1) and polycomb repressed (H3K27me3) regions. FOXA2 TFBS across these stages were also predicted using MACS2 with standard settings (apart from FDR<0.01 and including input control).

We also obtained Encode ChIP-seq transcription factor data from four Encode cell lines (EncodeProjectConsortium, 2012) representing cells derived from different germ layers. Specifically, these data represented stem cells (HESC1, TF=BCL11A Encode Experiment: ENCSR000BIP), endodermal (HepG2 cell line which is derived from a patient with liver carcinoma, TF=FOXA2, Encode Experiment: ENCSR000BNI), mesodermal (K562 cell line which is an immortalised cell line produced from a patient with chronic myelogenous leukemia, TF=NEUROD1, Encode Experiment: ENCSR986CDX) or ectodermal (HeLaS3 cell line which represents an immortalised cell line derived from a cervical cancer patient, TF=STATA3 Encode Experiment: ENCSR000EDC) lineage committment. We also made use of publicly available human islet TFBS data (FOXA2, MAFB, NKX2-2, NKX6-1, PDX1) from (Pasquali et al., 2014). FASTQ reads were processed and TFBS predicted based on MASC2 open chromatin peaks which were identified as described above.

To determine annotation enrichment, DOCS were again permuted (shuffled) 1000 times using bedtools to distribute them randomly across the genome. Enrichment was calculated by comparing the number of true DOCS overlapping a given annotation compared to the number of random DOCS overlapping the same annotation.

2.8 GREAT enrichment analysis

Bi-DOCS were tested for gene and pathway enrichment using GREAT (McLean et al., 2010) with standard settings. Pathways and GO terms with a minimum binomial and gene enrichment of 2 and an FDR < 0.05 were considered significant and reported with the associated genes. The enrichment of Bi-DOCS overlapping hESC (H1 cell line from Encode) chromatin states (including bivalent enhancer and bivalent promoter states) was determined by defining the foreground set as chromatin states (of a given type) overlapping Bi-DOCS while as background set all chromatin states (of a given type) were used. Enrichment was determined by comparing the foreground set to the background set using a hypergeometric test. As before, Pathways and GO terms with a minimum enrichment>2 and FDR<0.05 were determined as significant.

2.9 *In silico* validation

To assess the impact of genotype: Sample labels were randomized and mixed (3 BiPSCs and 2 FiPSCs versus 3 FiPSCs and 2 BiPSCs). The newly generated randomised sample sets were reanalysed using diffReps as described above so as to evaluate tissue of origin and genotype effects by comparing the number of true VS random DOCS.

To assess the impact of karyotype: samples with abnormal karyotypes were removed and the diffReps analysis repeated as before. Correlation between DOCS identified in all samples and DOCS identified in karyotypically normal samples was evaluated using R.

Enrichment of BiDOCS in stage endodermal regulatory genes: publicly available stage specific endodermal regulatory genes were obtained from (van de Bunt et al., 2016). Bi-DOCS regions and islet ATAC-seq open chromatin regions were linked to genes through GREAT (McLean et al., 2010) and enrichment of Bi-DOCS associated genes (versus islet open chromatin associated genes) in stage specific endodermal regulatory genes was determined using a fisher test.

2.10 ChIP-seq qPCR validation

Cells were treated with 1% formaldehyde for 10 minutes at room temperature before lysis in buffer containing: 10%SDS, 10mM EDTA pH8, and 50mM Tris-HCl pH 8.1 (10 minutes at 4 ^{O}C). Chromatin was sonicated to 200-500-bp fragments using Bioruptor® Plus sonication device (Diagenode). Sonicated DNA fragment sizes range was validated by agarose gel electrophoresis. Immunoprecipitation was performed overnight using anti-H3K4me3 antibody (Abcam ab8580) or normal rabbit IgG (Millipore 12-370). Crosslinking reversal was preformed overnight at 65°C with proteinase K (Thermo scientific EO0491)). DNA was recovered using a PCR clean-up kit (Qiagen 28004). Eluted DNA fragments were used for qPCR analysis with primers listed in Table S4.

Enrichment of open chromatin was validated using primers recognizing the APRT promoter (data not shown). Normal rabbit IgG was used to determine background levels.

2.11 iPSC differentiation

Four FiPSCs lines (from two human donors) and four BiPSCs lines (from three human donors) were induced to differentiate into definitive endoderm (DE) and pancreatic progenitors (PP), using a published protocol (Rezania et al., 2014). These eight iPSCs lines were the same lines used for the ATAC-seq analysis, and in the same range of passage number (Table S1). The differentiation was carried out in duplicates. RNA was collected at the end of each differentiation stage for RNA-seq analysis.

2.12 RNA-seq data processing and analysis

The TruSeq stranded paired-end RNA-seq libraries were prepared for a total of 32 samples and sequenced across 2 lanes of Illumina HiSeq2500 to a mean depth of 36.3 millions (±4.3 million) raw sequencing read pairs per sample. The sequencing quality was assessed with FASTQC (http://www.bioinformatics.babraham.ac.uk/projects/fastqc/) and the raw sequencing reads were mapped to the human genome hg19 using STAR version 2.5.1 (Dobin et al., 2013) with default settings. The GENCODE v19 GTF was applied to guide the spliced alignment. Duplicated alignments were marked with the MarkDuplicates script from the Picard tools v2.1.1 suite (http://broadinstitute.github.io/picard). Gene expression was quantified with featureCounts (Liao et al., 2014) using the GENCODE v19 GTF file. We restricted the downstream analysis to only the annotated protein-coding genes. Differential expression analysis between BiPSC and FiPSC at each differentiation stage was performed using DESeq2 R package (package v.1.10.1, under R v.3.2.5) (Love et al., 2014) using 5% FDR as the cut-off for reporting of the statistically significant results.

2.13 Differentially expressed gene list enrichment analysis

As previously described, we used GREAT (McLean et al., 2010) to assign genes to Bi-DOCS and Fi-DOCS regions, as well as the previously reported stage specific FOXA2 ChIP-seq peaks (Wang et al., 2015) at the definitive endoderm (DE) differentiation stage. We restricted the investigated FOXA2 targets to genes expressed at >1TPM in all samples at the DE stage. The significance of enrichment of genes upregulated in each lineage at the DE and PP stages within the Bi-DOCS, Fi-DOCS and FOXA2 targets genes lists was calculated using the upper tail of the hypergeometic distribution and the resulting p-values were adjusted for multiple testing using the Benjamini & Hochberg method of the p.adjust function within R v.3.2.5.

2.14 Statistical analysis and graphical visualisation

Statistical analysis (PCs, Spearman's rho and hierarchical clustering) and graphical visualisation was performed using R (version 3.0.2 (R-Core-Team, 2013)) and the ggplot2 package (Wickham, 2009).

2.15 Data access

Data has been deposited at the EBI hosted European Genome-phenome Archive (EGA, http://www.ebi.ac.uk/ega/) and European Nucleotide Archive (ENA, http://www.ebi.ac.uk/ena). EGA accession: EGAS00001002591 (islet ATAC-seq and iPSC RNA-seq). ENA accession: PRJEB21856 (iPSC ATAC-seq).

3. Supplemental References:

Buenrostro, J.D., Giresi, P.G., Zaba, L.C., Chang, H.Y., and Greenleaf, W.J. (2013). Transposition of native chromatin for fast and sensitive epigenomic profiling of open chromatin, DNA-binding proteins and nucleosome position. Nat Methods *10*, 1213-1218.

Cross, S.E., Hughes, S.J., Clark, A., Gray, D.W., and Johnson, P.R. (2012). Collagenase does not persist in human islets following isolation. Cell Transplant *21*, 2531-2535.

Dobin, A., Davis, C.A., Schlesinger, F., Drenkow, J., Zaleski, C., Jha, S., Batut, P., Chaisson, M., and Gingeras, T.R. (2013). STAR: ultrafast universal RNA-seq aligner. Bioinformatics *29*, 15-21.

Ernst, J., and Kellis, M. (2012). ChromHMM: automating chromatin-state discovery and characterization. Nat Methods *9*, 215-216.

Hay, D., Hughes, J.R., Babbs, C., Davies, J.O., Graham, B.J., Hanssen, L.L., Kassouf, M.T., Oudelaar, A.M., Sharpe, J.A., Suciu, M.C*., et al.* (2016). Genetic dissection of the alpha-globin super-enhancer in vivo. Nat Genet *48*, 895-903.

Langmead, B., Trapnell, C., Pop, M., and Salzberg, S.L. (2009). Ultrafast and memory-efficient alignment of short DNA sequences to the human genome. Genome Biol *10*, R25.

Li, H., Handsaker, B., Wysoker, A., Fennell, T., Ruan, J., Homer, N., Marth, G., Abecasis, G., Durbin, R., and Genome Project Data Processing, S. (2009). The Sequence Alignment/Map format and SAMtools. Bioinformatics *25*, 2078-2079.

Liao, Y., Smyth, G.K., and Shi, W. (2014). featureCounts: an efficient general purpose program for assigning sequence reads to genomic features. Bioinformatics *30*, 923-930.

Love, M.I., Huber, W., and Anders, S. (2014). Moderated estimation of fold change and dispersion for RNA-seq data with DESeq2. Genome Biol *15*, 550.

Magoc, T., and Salzberg, S.L. (2011). FLASH: fast length adjustment of short reads to improve genome assemblies. Bioinformatics *27*, 2957-2963.

McLean, C.Y., Bristor, D., Hiller, M., Clarke, S.L., Schaar, B.T., Lowe, C.B., Wenger, A.M., and Bejerano, G. (2010). GREAT improves functional interpretation of cis-regulatory regions. Nat Biotechnol *28*, 495-501.

Quinlan, A.R., and Hall, I.M. (2010). BEDTools: a flexible suite of utilities for comparing genomic features. Bioinformatics *26*, 841-842.

R-Core-Team (2013). R: A Language and Environment for Statistical Computing (VIenna, Austria: R Foundation for Statistical Computing).

Ramirez, F., Ryan, D.P., Gruning, B., Bhardwaj, V., Kilpert, F., Richter, A.S., Heyne, S., Dundar, F., and Manke, T. (2016). deepTools2: a next generation web server for deep-sequencing data analysis. Nucleic Acids Res *44*, W160-165.

Russ, H.A., Bar, Y., Ravassard, P., and Efrat, S. (2008). In vitro proliferation of cells derived from adult human beta-cells revealed by cell-lineage tracing. Diabetes *57*, 1575-1583.

Shen, L., Shao, N.Y., Liu, X., Maze, I., Feng, J., and Nestler, E.J. (2013). diffReps: detecting differential chromatin modification sites from ChIP-seq data with biological replicates. PLoS One *8*, e65598.

van de Bunt, M., Manning Fox, J.E., Dai, X., Barrett, A., Grey, C., Li, L., Bennett, A.J., Johnson, P.R., Rajotte, R.V., Gaulton, K.J*., et al.* (2015). Transcript Expression Data from Human Islets Links Regulatory Signals from Genome-Wide Association Studies for Type 2 Diabetes and Glycemic Traits to Their Downstream Effectors. PLoS genetics *11*, e1005694.

Wickham, H. (2009). ggplot2: Elegant Graphics for Data Analysis (New York: Springer-Verlag).

Zhang, Y., Liu, T., Meyer, C.A., Eeckhoute, J., Johnson, D.S., Bernstein, B.E., Nusbaum, C., Myers, R.M., Brown, M., Li, W*., et al.* (2008). Model-based analysis of ChIP-Seq (MACS). Genome Biol *9*, R137.
